# Uncovering memory-related gene expression in contextual fear conditioning using ribosome profiling

**DOI:** 10.1016/j.pneurobio.2020.101903

**Published:** 2021-02

**Authors:** Konstanze Simbriger, Inês S. Amorim, Gilliard Lach, Kleanthi Chalkiadaki, Stella Kouloulia, Seyed Mehdi Jafarnejad, Arkady Khoutorsky, Christos G. Gkogkas

**Affiliations:** a Centre for Discovery Brain Sciences, University of Edinburgh and Patrick Wild Centre and Simons Initiative for the Developing Brain, University of Edinburgh, EH8 9XD, Edinburgh, Scotland, UK; b Patrick G. Johnston Centre for Cancer Research and Cell Biology, The Queen’s University of Belfast, BT9 7AE Belfast, Northern Ireland, UK; c Department of Anesthesia, Faculty of Dentistry and Alan Edwards Centre for Research on Pain, McGill University, H3A 0G1, Montréal, QC, Canada

**Keywords:** Fear conditioning, Translational profiling, Memory

## Abstract

Contextual fear conditioning (CFC) in rodents is the most widely used behavioural paradigm in neuroscience research to elucidate the neurobiological mechanisms underlying learning and memory. It is based on the pairing of an aversive unconditioned stimulus (US; e.g. mild footshock) with a neutral conditioned stimulus (CS; e.g. context of the test chamber) in order to acquire associative long-term memory (LTM), which persists for days and even months. Using genome-wide analysis, several studies have generated lists of genes modulated in response to CFC in an attempt to identify the “memory genes”, which orchestrate memory formation. Yet, most studies use naïve animals as a baseline for assessing gene-expression changes, while only few studies have examined the effect of the US alone, without pairing to context, using genome-wide analysis of gene-expression. Herein, using the ribosome profiling methodology, we show that in male mice an immediate shock, which does not lead to LTM formation, elicits pervasive translational and transcriptional changes in the expression of Immediate Early Genes (IEGs) in dorsal hippocampus (such as *Fos* and *Arc*), a fact which has been disregarded by the majority of CFC studies. By removing the effect of the immediate shock, we identify and validate a new set of genes, which are translationally and transcriptionally responsive to the association of context-to-footshock in CFC, and thus constitute salient “memory genes”.

## 1 Main

Pavlovian fear conditioning, which involves learning that environmental stimuli can predict aversive events, is perhaps the most widely used behavioural paradigm in neuroscience research to elucidate the neurobiological mechanisms underlying learning and memory (Maren et al., 2013). In contextual fear conditioning (CFC) in rodents, an aversive unconditioned stimulus (US; e.g. mild footshock) is paired with a conditioned stimulus (CS; context of a test chamber) to form an associative long-term memory (LTM) between the context and footshock, which can last for many days and even months. Upon re-exposure to the context (CS), the memory is retrieved and manifested in prey animals like rodents as freezing behaviour (Fanselow, 1980). LTM formation in the CFC task depends on changes in gene expression in the hippocampus and there is evidence that the dorsal hippocampus is strongly linked to the formation and consolidation of memory. With the advent of genome-wide gene expression analysis technologies (such as microarray and more recently RNA sequencing), several studies have generated lists of genes modulated in response to CFC, in an attempt to identify the “memory genes”, which orchestrate memory formation. However, in the majority of these genome-wide studies there is omission of the important control for the effect of the US alone on modulation of gene-expression, as naïve animals were predominantly used as the baseline for assessing gene-expression changes (compare Sup. Table 1). Several single-gene studies (e.g. on *Fos* and *Arc*), however, include naïve mice and mice exposed to US only (unpaired) as control groups (Cho et al., 2017; Rosen et al., 1998). Therefore, it is crucial to ascertain which genes are modulated during CFC, by assessing the effect of the US alone on gene-expression, using a high-resolution, genome-wide methodology.

**Fig. 1:**
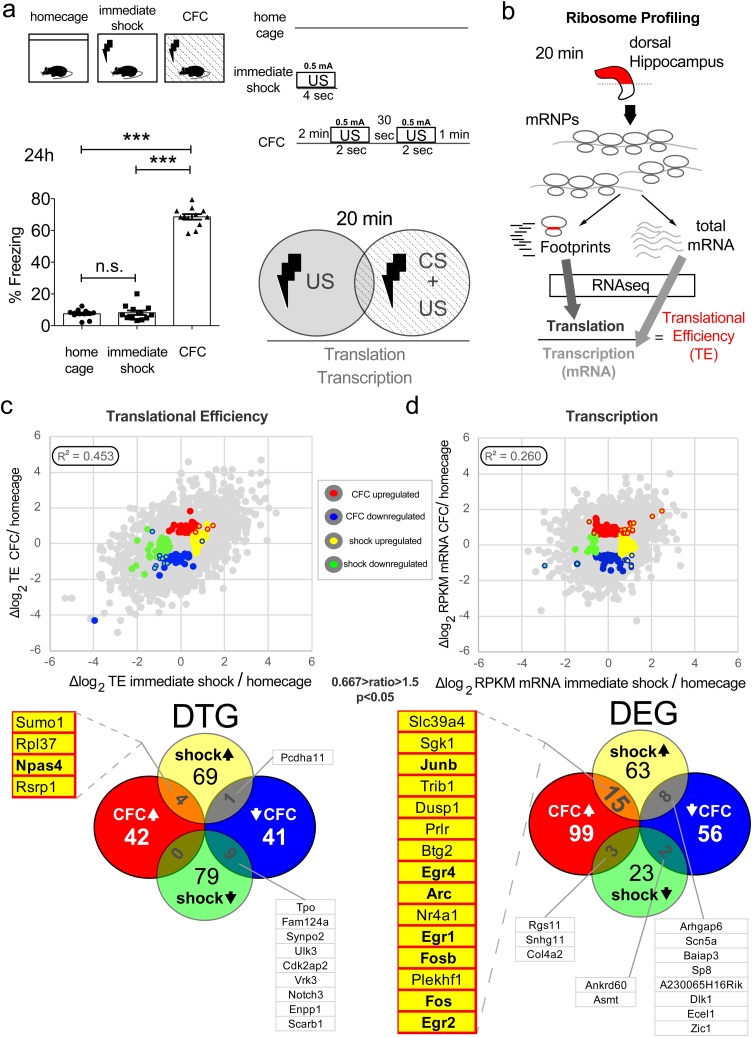
Profiling translational and transcriptional changes in mouse dorsal hippocampus 20 min following a Contextual Fear Conditioning paradigm. Comparison of the unconditioned stimulus alone (US; footshock) versus the pairing of Conditional Stimulus (CS; context) with US (CS + US). **a.** TOP: Schematic illustration of the behavioural paradigm design with three groups: *homecage*, *immediate shock* (US; 0.5 mA, 4 s) and *CFC* (CFC; CS + US; 2 min exploration of the chamber followed by two footshocks (0.5 mA, 2 s duration) with 30 s inter-shock interval, followed by 1 min resting in the chamber). BOTTOM: Percentage freezing of mice 24 h after CFC for the three groups depicted. One-way ANOVA with Tukey’s post-hoc; n = 12 mice per group; ***p < 0.001. **b.** Graphic outlining of the mRNA-Seq and ribosome profiling strategy used to measure genome-wide translational and transcriptional changes in dorsal hippocampus mouse tissue isolated 20 min post-CFC; mRNP: messenger ribonucleoprotein **c.** TOP: Scatter plot and Pearson correlation (R^2^) of translational efficiency (TE; footprint RPKM normalised to mRNA RPKM) and of **d.** RPKM between *CFC* and *immediate shock* dorsal hippocampus, both normalised to *homecage*. Differentially translated (DTGs) or expressed (DEGs) genes are depicted with different colours corresponding to the groups analysed. BOTTOM: Venn diagrams of individual and overlapping DEGs and DTGs between experimental groups. Lists of DEGs and DTGs, which correspond to IEGs and are discussed in the text are highlighted; cut-off used: 0.667 > ratio>1.5. See also Supplementary Figure 1, 2 and Supplementary Table 2.

First, to assess the contribution of US alone, we designed a CFC paradigm which included three experimental groups: “homecage” (naïve mice without exposure to footshock or the context), “immediate shock” (mice that were not allowed to pair the context to footshock: no exploration → 1 footshock of 4 s → removed immediately upon shock termination) and “CFC” (mice that were allowed to pair the context to the footshock: 2 min exploration → 2 footshocks of 2 s with 30 s interval → 1 min post-shock stay in the box) (Fig. 1a; top). All mice tested in this study were adult (10-week-old) males. Examination of LTM revealed that only animals in the *CFC* group displayed ∼70 % freezing behaviour 24 h post-CS + US pairing, whereas *immediate shock* was not sufficient to evoke memory formation (Fig. 1a; bottom left). Second, to study the early genome-wide transcriptional and translational changes in *CFC* (20 min post-training), with high resolution, we implemented an mRNA-Seq and ribosome profiling strategy (Fig. 1b). Ribosome-protected fragments (footprints; a proxy for translation) and total mRNA fragments (a proxy for transcription) were extracted from dorsal hippocampus tissue (Fig. 1b), aiming to measure genome-wide translational efficiency (TE). High quality polysomes, which are crucial for downstream analysis, were isolated in our samples using sucrose gradient polysome profiling (Sup. Fig. 1a). Previous studies reported low-quality polysomes (using polysome profiling) in hippocampal tissue and reduced TE of ribosomal proteins coding genes (using ribosome profiling), which were subsequently claimed to be brain-specific and not observed in other tissues or cell types, such as mouse Embryonic Stem Cells (mESCs) (Cho et al., 2015). We found that compared to other cell types, dissociated cultured hippocampal neurons and dorsal hippocampus lysates contain prominent light and heavy polysomes and a reduced 80S monosome peak (Sup. Fig. 1a). Moreover, contrary to a previous report (Cho et al., 2015), we did not observe a dramatic and uniform reduction in the expression of all ribosomal proteins in hippocampal tissue compared to other mouse tissues examined (kidney, muscle, spleen) (Sup. Fig. 1b). We compared TE measurements from our study with several studies measuring TE in various tissues for all protein-coding mRNAs, ribosomal protein-coding mRNAs and mRNAs encoding mitochondrial ribosomal proteins. We observed that in all examined tissues, there is a trend for reduced TE for ribosomal protein-coding mRNAs, as compared to all proteins, to mitochondrial ribosomal proteins or to other mRNAs, which have similar length to ribosome protein coding mRNAs (Sup. Fig. 1c). However, the decrease in TE seen in hippocampus for ribosomal protein coding mRNAs was comparable to other tissues. Together, these data reveal that neuronal cells and tissue contain abundant polysomes and do not display hippocampus/neuron-specific repression of translation of ribosomal protein-coding mRNAs.

Using ribosome profiling, we measured genome-wide RPKM (Reads per Kilobase of transcript per Million mapped reads) for footprint and total mRNA libraries (Fig. 1c, d). HiSeq2500 produced high quality reads for both types of libraries, as evidenced first by the canonical distribution of footprint size (28–32 nt) (Sup. Fig. 2a), second by the read distribution within the three reading frames (Sup. Fig. 2b), third by the canonical periodicity of ribosomal footprints across mRNA coding and untranslated regions (Sup. Fig. 2c), fourth by the R^2^ of RPKM between biological replicates (n = 2) for the three experimental groups, which is >0.98 for both footprints and total mRNA (Sup. Fig. 2d), and fifth by the principal components analysis of biological replicates (Sup. Fig. 3). *CFC* and *immediate shock* engendered pervasive translational and transcriptional changes in dorsal hippocampus 20 min after terminating the respective protocol, as compared to *homecage* group animals (Fig. 1c, d). This is evidenced by the low correlation (Pearson R^2^<0.45) of log_2_ TE or RPKM normalised to *homecage*, for translation and transcription respectively (Fig. 1c, d). We then established differentially translated (DTGs) and transcribed (DEGs) genes, upregulated or downregulated for *CFC* or *immediate shock* conditions (Fig. 1c, d and Sup. Table 1). In accordance with previous studies, we observed a robust induction of IEGs 20 min post-CFC (Cho et al., 2015; Alberini and Kandel, 2014). However, while there is a significant number of *CFC*-specific or *immediate shock*-specific DTGs and DEGs, we also identified an overlap between *CFC* and *immediate shock* categories for IEGs in DTGs (*Npas4*; Fig. 1c) and DEGs (*Egr2, Fos, Fosb, Egr1, Arc, Egr4* and *Junb*; Fig. 1d). Thus, both *immediate shock*, a stimulus which did not produce memory (Fig. 1a bottom), and *CFC*, which elicited contextual fear memory (Fig. 1a bottom), induced activation of several IEGs mainly at the level of mRNA. This constitutes a major confound for reporter systems based on such IEGs (mainly Fos and Arc) designed to capture neuronal ensembles (Barth, 2007), which are used to study different types of hippocampus-dependent memories. In addition, the majority of genome-wide gene expression studies using CFC do not include the *immediate shock* condition as a control (Sup. Table 1). Moreover, a recent study using cell-type-specific profiling identified *Npas4* as a key translational target in CFC in the hippocampus (Eacker et al., 2017), while previous literature has highlighted the importance of *Npas4* in acquiring different types of contextual memory (Heroux et al., 2018) and as a transcriptional regulator in CFC (Ramamoorthi et al., 2011).

**Fig. 2:**
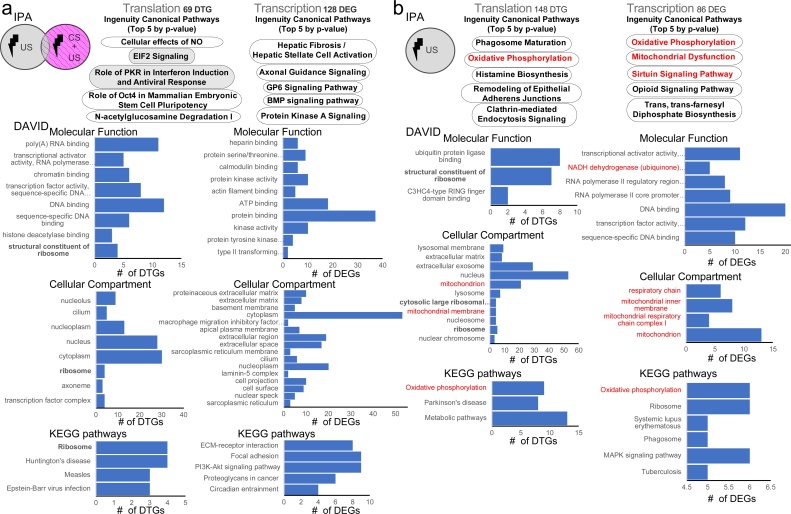
Ingenuity Pathway Analysis and DAVID Gene Ontology of DEGs and DTGs from *CFC* minus *immediate shock* and *immediate shock* groups. TOP: IPA Canonical pathway analysis showing the top 5 categories for DTGs (Translation) and DEGs (Transcription) for the *CFC* minus *immediate shock* group **a.** and the shock only group **b.** BOTTOM: For both **a.** and **b.** DAVID analysis showing Molecular Function, Cellular Compartment and KEGG Pathways GO categories in ascending p-value order (Bonferroni’s post-hoc). Categories discussed in the text are highlighted. See also Supplementary Table 3.

We next used an unbiased approach, combining Ingenuity Pathway Analysis (IPA) and the Database for Annotation, Visualization and Integrated Discovery (DAVID) to identify Gene Ontology (GO) categories and molecular/cellular pathways, affected either in the *CFC* group (after removing the overlapping genes with the *immediate shock* group, “*CFC* minus *immediate shock*”) or in the *immediate shock* group (Fig. 2a, b and Sup. Table 2). First, using IPA and DAVID analyses for the two groups examined (*CFC* minus *immediate shock* and *immediate shock*), there was only overlap in GO categories and pathways associated with ribosomes (Fig. 2a, b). Second, for the *CFC* group, we detected among the top 5 canonical IPA pathways, eukaryotic translation initiation factor 2 (eIF2), a gene previously associated with learning and memory (Costa-Mattioli et al., 2007), and recently shown to be important for contextual fear memories using neuron-specific profiling with TRAP (translating ribosome affinity purification (Eacker et al., 2017)). Third, in *CFC* minus *immediate shock* DTGs, we observed regulation of GO categories linked to transcriptional and DNA regulation (such as chromatin regulation and transcription factors). Fourth, in the *immediate shock* group, we observed with both IPA and DAVID robust modulation of pathways associated with mitochondria (such as oxidative phosphorylation and mitochondrial dysfunction; Fig. 2b). Taken together, these data suggest that *immediate shock* activates distinct cellular pathways, which differ significantly from the *CFC*-modulated pathways during memory acquisition.

**Fig. 3:**
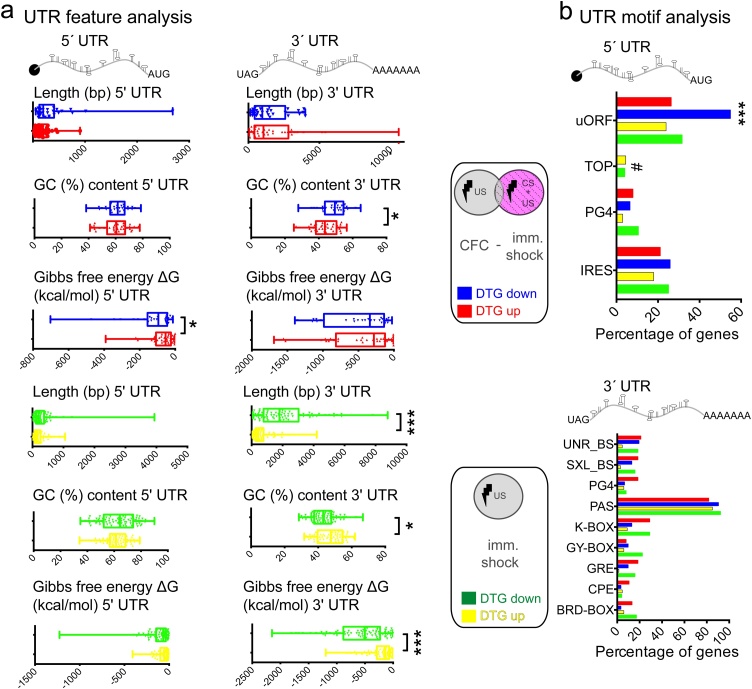
UTR analysis of DEGs and DTGs from *CFC* minus *immediate shock* and *immediate shock* groups. Analysis of DTG UTRs **a.** 5′ and 3′ UTR Length (in bp), GC% content and Gibbs Free Energy predicted by mFold (kcal/mol) are shown; ***p < 0.001, **p < 0.01, Student’s *t*-test **b.** Percentage of DTGs containing the depicted 5′ or 3′ UTR motifs, using UTRscan and UTRdb. ***p < 0.001, Two-way ANOVA with Bonferroni’s post-hoc; 5′ UTR: motif type F (3, 9) = 16.37; p = 0.0005, experimental group F (3, 9) = 1.373; p = 0.312, 3′ UTR: motif type F (8, 24) = 102.8; p < 0.0001, experimental group F (8, 24) = 9.376; p > 0.999. Red: *CFC* minus *immediate shock* Upregulated DTGs; Blue: *CFC* minus *immediate shock* Downregulated DTGs; Yellow: *immediate shock* minus *CFC* Upregulated DTGs, Green: *immediate shock* minus *CFC* Downregulated DTGs. # denotes the presence of TOP motifs only in *CFC* groups.

Given the distinct translatomes modulated by *CFC* and *immediate shock*, we reasoned that 5′ or 3′ untranslated region (UTR) features (structure, sequence) of DTG mRNAs may explain their preferential translational modulation (Hinnebusch et al., 2016; Jung et al., 2014). Thus, we examined length, Guanine-Cytosine (GC%) content and calculated the free energy (Gibbs; kcal/mol) required to dissolve secondary structures in mRNA sequences, as predicted by the mFold software (Zuker, 2003) (Fig. 3). In *CFC* DTGs, we detected less complex 5′ UTRs and decreased GC content in 3′ UTRs of upregulated DTGs (Fig. 3a; top). In *immediate shock* DTGs, we found no changes in 5′ UTRs, but shorter, with higher GC content and less complex 3′ UTRs in upregulated DTGs (Fig. 3a; bottom). These results suggest that contextual memory in *CFC* is acquired following translational control of a subset of genes, via 5′ UTR-related mechanisms, and thus possibly involving translation initiation control. To further elucidate the mechanisms linked to mRNA UTRs, which are implicated in contextual memory acquisition, we employed UTRscan and the database of UTR motifs UTRdb (Grillo et al., 2010) and analysed the 5′ or 3′ UTR sequences of *CFC* and *immediate shock* DTGs and detected several known motifs implicated in translational control (Fig. 3b). First, we observed a statistically significant (Two-way ANOVA with Bonferroni’s post-hoc) increase in upstream open reading frame (uORF)-containing 5′ UTRs in the *CFC* downregulated DTGs, as compared to all other groups (p < 0.0001) and detected terminal oligopyrimidine tract containing (TOP) mRNAs in *immediate shock* DTGs, but not in *CFC* (Fig. 3b; top). While we observed a significant effect for motif type, there were no significant changes (Two-way ANOVA with Bonferroni’s post-hoc) in 3′ UTR motifs between *CFC* and *immediate shock* DTGs (Fig. 3b; bottom). Contextual memory acquisition by CFC was shown to reduce eIF2α phosphorylation in the hippocampus (Costa-Mattioli et al., 2007) and its importance was further highlighted with neuron-specific TRAP (Eacker et al., 2017). High levels of eIF2α phosphorylation preferentially stimulate translation of uORF-containing genes (such as ATF4) (Costa-Mattioli et al., 2007). The enrichment of uORF-containing *CFC* downregulated genes is in line with reduced eIF2α phosphorylation following CFC (Costa-Mattioli et al., 2007), suggesting that it constitutes a specific response to acquisition of contextual memory and is in agreement with previous work (Eacker et al., 2017).

This result (Fig. 3b), in conjunction with the eIF2-related GO categories we identified in *CFC*-specific DTGs using unbiased IPA analysis (Fig. 2), supports the key role of eIF2 signalling in the hippocampus during contextual fear memory acquisition and furthermore bolsters its significance as it is not modulated by *immediate shock*.

**Fig. 4:**
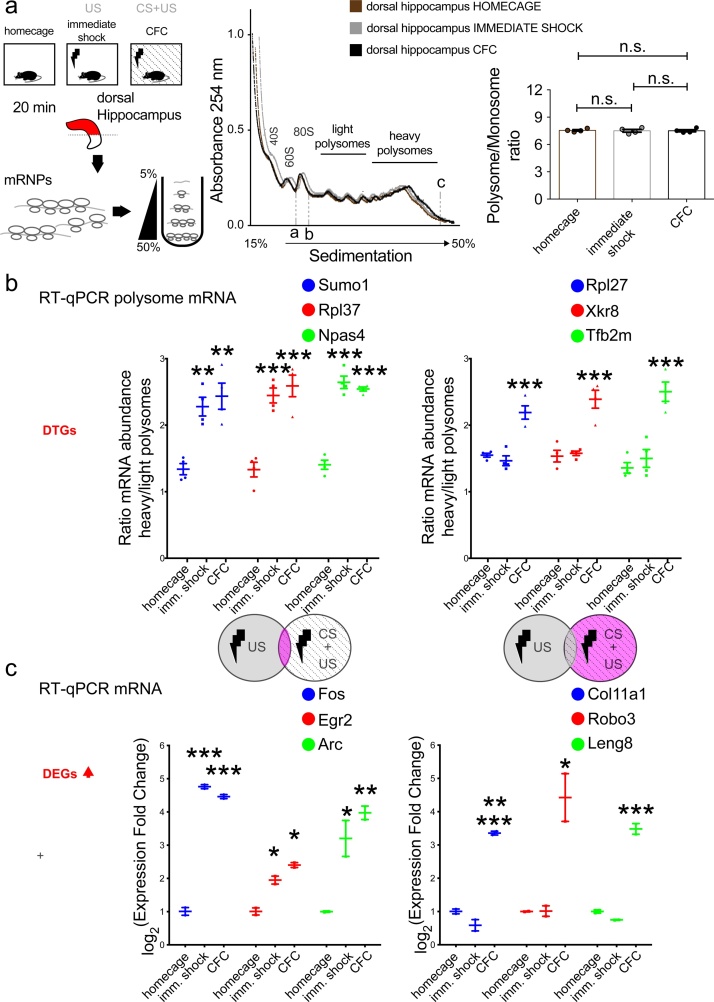
Validation of DTGs and DEGs discovered with ribosome profiling. a. Polysome profiling analysis of lysates from dorsal hippocampus dissected 20 min post-learning for the indicated groups (*homecage*, *immediate shock*, *CFC*). Continuous UV absorbance at 254 nm of lysates resolved over a 5–50 % sucrose gradient. 40S, 60S, 80S (monosome), light and heavy polysomes are marked on the absorbance graph. Polysome/Monosome ratio for all groups was calculated as the fraction of the area under the curve between b→c (polysomes) over the area between a→b (monosome; 80S); n.s. not significant, Student’s *t-*test. **b.** RT-qPCR from total mRNA extracted from light and heavy polysome fractions for genes upregulated in both *immediate shock* and *training* (left) and genes upregulated in the *training* but not the *immediate shock* group (right), with specific primers for the indicated genes. Ratio of mRNA abundance is shown; n = 4 biological replicates (2–3 animals per replicate) per group, **p < 0.01, One-way ANOVA with Bonferroni’s post-hoc **c.** RT-qPCR from total mRNA extracted from dorsal hippocampus lysates for genes upregulated in both *immediate shock* and *training* (left) and genes upregulated in the *training* but not the *immediate shock* group (right), with specific primers for the indicated genes. Log_2_ of expression fold change is shown; n = 2 biological replicates (5–6 animals per replicate) per group. ***p < 0.01, **p < 0.01, *p < 0.05; One-way ANOVA with Bonferroni’s post-hoc.

To further validate the findings of the ribosome profiling assay in identifying DTGs and DEGs, which are specific to the CS + US pairing, but not to the *immediate shock*, we carried out polysome profiling of dissected dorsal hippocampus lysates for the 3 experimental groups (*homecage*, *immediate shock* and *CFC*) 20 min post-stimulation (Fig. 4a; left). Resolving lysates on a sucrose gradient revealed no significant changes in overall polysome profiles, as evidenced by the polysome/monosome ratio (Fig. 4a; right). We purified polysome-associated mRNAs and carried out RT-qPCR with specific primers for the top three DTGs to measure their mRNA abundance in heavy versus light polysomes (Fig. 4b). We found that the translation of *Sumo1*, *Rpl37*, and *Npas4* mRNAs was upregulated both in *immediate shock* and *CFC* conditions, whereas translation of *Rpl27*, *Xkr8* and *Tfb2m* mRNAs was upregulated only in *CFC*, as compared to *homecage* (Fig. 4b). Likewise, we used total mRNA from the three experimental groups and performed RT-qPCR with specific primers for the top three DEGs, measuring their abundance (Fig. 4c). We found that expression of the DEGs (and IEGs) *Fos, Egr2* and *Arc* was upregulated both in *immediate shock* and *CFC*, while *Col11a1, Robo3* and *Leng8* were upregulated only in *CFC*, as compared to *homecage* (Fig. 4c). Taken together, these data further validate our ribosome profiling-detected DTGs and DEGs and provide a set of translationally and transcriptionally regulated salient “memory genes”. Interestingly, *Col11a1* is differentially expressed in the superficial-deep CA1 hippocampal axis, linked to hippocampal place cells and spatial memory (Mallory and Giocomo, 2018; Cembrowski et al., 2016), *Robo3* encodes a receptor with specificity in the mammalian lineage and is a key player in neural development (Friocourt and Chedotal, 2017), while *Leng8* was previously shown to be upregulated in mouse hippocampus 1 h after CFC (Peleg et al., 2010). These newly identified genes are predicted by our genome-wide analysis to be highly relevant to contextualization of fear memory, suggesting that they may also be relevant to other forms of hippocampus-dependent learning and memory.

Notably, our study has several limitations. First, we used different stimulation protocols for *CFC* (two 2 s footshocks with 30 s interval) and *immediate shock* (4 s footshock). Studies such as Bernier et al. (Bernier et al., 2014) have shown how post-shock intervals as short as 30 s can lead to fear memories, therefore, the continuous 4 s footshock in the *immediate shock* condition was used to avoid pairing of footshock with context during the 30 s interstimulus interval. Second, we examined the effect of footshock alone (without pairing to context) on gene expression; however, the effect of context alone (without pairing to footshock) has not been evaluated. This important control group should be included in future studies. Third, all our analyses were performed 20 min post-learning. This time point prioritises the detection of alterations in IEGs at the transcriptional level and might not capture the full repertoire of changes in gene expression (transcription and translation) occurring at earlier or later time points (Cho et al., 2015).

We show that expression of IEGs (such as Fos, Arc and Egr1), which have been widely used in neuroscience research to identify activated neuronal cells relevant to memory, is modulated both in *CFC* and *immediate shock* conditions, suggesting that their induction may misrepresent true “memory neurons”. This conclusion is supported by several previous studies showing non-specific induction of these genes by US alone (Cho et al., 2017; Rosen et al., 1998; Peter et al., 2012), but not by other reports demonstrating selective stimulation of their expression by associative learning and not US (Jarvis et al., 1995). These discrepancies might be related to differences in experimental design of learning paradigms and detection methods. Importantly, previous studies have established central roles of Fos (Fleischmann et al., 2003; Guzowski, 2002; Kemp et al., 2013), Arc (Plath et al., 2006; Guzowski et al., 2000; Peebles et al., 2010) and Egr1 (Jones et al., 2001; Bozon et al., 2003) in memory consolidation as their deletion impairs different forms of long-term memory. Arc is involved in activity-dependent formation of new synapses and dendritic reconfiguration (Nikolaienko et al., 2018), whereas Egr1 controls the expression of late-response genes involved in growth and synaptic plasticity (Duclot and Kabbaj, 2017; Koldamova et al., 2014). How Fos regulates memory formation remains largely unknown. Conceivably, different levels of transcriptional or translational activation of specific genes could be the mechanism by which the brain would distinguish between stimuli corresponding to our experimental groups; *immediate shock and* CFC, in order to achieve the formation of a specific memory trace. We did not detect any significant differences in fold change of transcriptional or translational activation for the top genes identified in this study (Fig. 4 and Sup. Fig. 4). Possibly, examination of additional timepoints comparing *immediate shock* to *CFC* would validate or disprove this proposed mechanism. Translation can be uncoupled from transcription (translational buffering), highlighting the importance of measuring translation or protein levels and not relying solely on changes in mRNA expression. Herein, using ribosome profiling we measure transcriptional and translational changes in brain, genome-wide, using a paradigm which is highly relevant for contextual fear memory formation.

In summary, using a CFC paradigm, we identified a list of salient “memory genes” (at the level of translation and transcription), and dissected the genome-wide effect of *immediate shock* and of the CS + US pairing, on gene expression, genome-wide. Moreover, we identified distinct 5′ UTR features of *CFC*-induced mRNAs and validated new gene markers that may be used to monitor cell activation in the CFC paradigm, with high specificity.

## 2 Methods

### 2.1 Mice

All procedures were in accordance with United Kingdom Home Office and Canadian Council on Animal Care regulations and were approved by the University of Edinburgh and McGill University. Animals were kept under standard husbandry conditions, with *ad libitum* access to food and water, unless otherwise specified. The animal facility was operated on a 12 h light/dark cycle. Wild-type mice were C57BL/6 J. We used 10-week old males for all groups. For ribosome profiling we used 2 biological replicates, each containing dorsal hippocampi from 5 to 6 animals – total RNA was also used for RT-qPCR. For polysome profiling we used 4 biological replicates (n = 2–3 animals per replicate).

### 2.2 Contextual fear conditioning and immediate shock paradigms

For ribosome profiling, we used 3 groups of animals: *homecage* (animals that did not receive a footshock or exposure to the context; remained in the homecage but were transported together with experimental animals to the experimental room), *immediate shock* (animals that were placed in the training box for the duration of a 4 s-footshock and removed immediately upon its termination) and *CFC* (animals that were allowed to pair the context to the footshock by initially exploring the training box for 2 min, by receiving 2 footshocks; 2 s duration and 30 s apart, and by remaining in the training box post-shock for 1 min). Animals from *CFC* and *immediate shock* were returned to their homecage after the procedure and 20 min after their individual protocols were sacrificed, bilateral hippocampi were removed, and the dorsal hippocampus dissected and flash frozen in liquid nitrogen.

For groups tested for LTM (n = 12 animals per group; *homecage*, *immediate shock*, *CFC*), 24 h after training, mice were tested for contextual fear memory, as assessed by % freezing in the conditioning context for a 5 min period, in 5 s intervals, either “freezing” or “not freezing”. Freezing (%) indicates the number of intervals where freezing was observed divided by the total number of 5 s intervals.

### 2.3 Ribosome profiling and bioinformatics analysis

We used the Epicentre TruSeq Ribo Profile (Mammalian) Kit (Illumina, RPYSC12116), with some modifications, to generate sequencing libraries. In brief, polysomes were extracted from snap-frozen, dorsal hippocampal tissue, pooled from 5–6 animals per condition, in the presence of Cycloheximide. A partial volume of these lysates was digested with TruSeq Ribo Profile Nuclease (Ribosome Protected Fragments, RPF), while another part of the lysate was kept as an internal transcription control (Total RNA). After digestion, RPFs were purified on MicroSpin S-400 columns as described in the kit to enrich for small RNA fragments (28−30 nt). All samples (RPF and Total RNA) were depleted of ribosomal RNA using the Ribo-Zero Gold (Human/Mouse/Rat) Kit (Illumina, MRZG126). RPFs only were purified on a 15 % TBE-Urea polyacrylamide gel, selecting bands running between 28 and 30 nt. Only Total RNA samples were heat fragmented. All samples were end-repaired using TruSeq Ribo Profile Polynucleotide kinase, followed by ligation of a TruSeq Ribo Profile 3′ Adapter. All samples were reverse transcribed into cDNA, followed by a further PAGE purification on a 10 % TBE-Urea gel, to separate sample cDNA from excess adapter. Purified cDNA was circularized and PCR amplified and afterwards purified using the Agencourt AMPure XP kit (Beckman Coulter). To increase the quantity and concentration of our libraries, we ran several PCR reactions in parallel and pool-purified the reactions using the Agencourt AMPure XP kit. PCR products were further purified on a 8 % TBE polycrylamide gel, to yield sufficient quantity and quality for sequencing. All samples were analysed on an Agilent Bioanalyzer High Sensitivity DNA chip to confirm expected size range and quantity and sequenced on an Illumina HiSeq 2500 system. The sequencing data was de-multiplexed by the sequencing facility (Edinburgh Genomics). Obtained sequences were analysed using a custom developed pipeline (following the methods used by Ingolia et al. (Ingolia et al., 2011)*)*. In brief, reads were adapter-trimmed using the FASTX toolkit, contaminant sequences (rRNA, tRNA) removed using bowtie and reads aligned to a reference genome using STAR. Cufflinks was used to quantify reads and calculate RPKM values for each transcript. Translational efficiency for each transcript was calculated by dividing RPKM values of the RPF libraries by RPKM values of the Total RNA libraries. Changes in transcription were analyzed for pairwise comparisons, based on experimental design, using microarray normalization methods, as reviewed by Quackenbush (Quackenbush, 2002). Changes in translation were assessed using the R package Xtail v1.1.5 (Xiao et al., 2016).

For further analysis of other published studies and of our sequencing (Sup Fig. 1c), transcripts similar in length to ribosomal protein coding transcripts, that were analysed for TE, were chosen by first defining the size range of ribosomal protein coding transcripts and then selecting all protein coding genes from our data that were within this size range. TE was then extracted for these genes from the analysis files and plotted along with the TE of all protein coding genes, ribosomal protein coding genes, and mitochondrial ribosomal protein coding transcripts.

### 2.4 Principal components analysis (PCA)

PCA was previously described in ref. (Amorim et al. (2018)) conducted with R package vegan version 2.4.4. Genes with undefined log2-transformed values (for RPKM 0 or TE 0) were excluded from the analysis. log2-transformed values of the remaining set of genes were standardized on a per-gene basis (scaled to mean 0 and SD 1). Euclidean distances of samples (replicates) were calculated from the same standardized log2-transformed gene data used in PCA. Hierarchical clustering based on the complete-linkage algorithm was performed on the distance matrix with R package stats version 3.4.2.

### 2.5 UTR analysis

UTR analysis of DTGs was carried out using a custom implemented pipeline that utilizes several publicly available tools. First, longest UTR sequences for each supplied gene ID were extracted from a database and basic statistics, such as length and guanine-cytosine (GC) content were extracted for each sequence. Gibbs free energy was calculated using mfold v3.6 (Zuker, 2003). Lastly, all sequences were scanned for known UTR motifs, using a stand-alone version of Utrscan (Grillo et al., 2010).

### 2.6 Gene Ontology and Pathway Analysis

Gene Ontology (GO) and Pathway Analysis were performed using, respectively, the online tool DAVID (Database for Annotation, Visualization and Integrated Discovery, version 6.8) and the Ingenuity Pathway Analysis Software (IPA; Qiagen Inc.). Datasets were uploaded on IPA and submitted to Core Analysis with analysis parameters set to include Direct and Indirect Interactions and Experimentally Observed data only. Ingenuity Canonical Pathways were obtained for all datasets and processed according to p-value. For GO analysis, datasets were submitted to DAVID and GO annotation gathered for KEGG pathways and Molecular Function and Cellular Component Gene Ontology Annotations. All raw output is summarised in Supplementary Table 3.

### 2.7 Polysome profiling

Polysome Profiling was carried out as previously described in ref. Gkogkas et al. (2013) with modifications. Dorsal hippocampi were rapidly dissected at the indicated times for each condition, washed with ice-cold PBS containing 100 μg/ml cycloheximide and flash-frozen in liquid N_2._ Using a pestle and mortar, tissue was pulverized on dry ice and the powder was resuspended in a hypotonic lysis buffer (5 mM Tris−HCl (pH 7.5), 2.5 mM MgCl_2_, 1.5 mM KCl, 100 μg/ml cycloheximide, 2 mM DTT, 0.5 % Triton X-100, and 0.5 % sodium deoxycholate). Lysate concentration was double balanced for protein: by using a Bradford-assay (BIORAD) and for RNA: by measuring total RNA concentration using a NANODROP2000 spectrophotometer (Thermo Scientific). Lysates were loaded onto 5–50 % sucrose density gradients (20 mM HEPES-KOH (pH 7.6), 100 mM KCl, 5 mM MgCl_2_) and centrifuged at 35,000 rpm for 2.5 h at 4 °C. The optical density (OD) at 254 nm was continuously recorded using an ISCO fractionator (Teledyne ISCO; Lincoln, NE) for each polysomal fraction; after extraction 5 ng of polyA + synthetic luciferase mRNA (Promega) was added to each fraction for subsequent balancing. Polysome to monosome ratio was calculated as the area under the A254 absorbance curve, using the function describing the recorded values, processed with the definite integral command in MATLAB.

### 2.8 RT-qPCR on polysomal RNA

Fractions for light and heavy polysomes for the indicated groups (n = 4) were pooled where indicated or processed separately, after balancing total RNA, measured with NANODROP2000 spectrophotometer (Thermo Scientific). Total RNA was isolated using Trizol (Invitrogen) and reverse transcribed using the Superscript III kit (Invitrogen) using a 1:1 mixture of oligo(dT) and random hexamers. cDNA was analyzed using a Biorad iQ SYBR Green Supermix kit as previously described in ref. Gkogkas et al. (2013) first for firefly luciferase expression to further balance cDNA. Results are presented as the ratio of heavy/light polysome mRNA abundance and were calculated in arbitrary units normalized to total RNA and to firefly luciferase RNA. Serial dilutions of cortical or hippocampal RNA were used as RT-qPCR concentration standards. The longest isoform for each gene was used to design RT-qPCRprimers with Primer-BLAST. The following primers were used: *Sumo1*: forward 5′-GGGTGAATCCACGTCACCAT-3′, reverse 5′-AGGAAAGCTCCCATTGGTCG-3′; *Rpl37*: forward 5′-TTGCTCTGGGATCCTACGCT-3′ reverse 5′-TCTAGCAAGCCTGCTCGTTC-3′; *Npas4*: forward 5′-ATCAGTGACACGGAAGCCTG-3′ reverse 5′-CTTGCTCAGGTCTGCTTGGA-3′; *Rpl27*: forward 5′-TTCAAAAACGCAGTGCCCGA-3′ reverse 5′−CCGGGTTTCATGAACTTGCC-3′; *Xkr8*: forward 5′−CCCTGGCATACAAATGTGGG-3′ reverse 5′-AACAAACCACGCAGACTCCA-3′; *Tfb2m*: forward 5′-AATCCTGACTGGGGCATTACT-3′ reverse 5′- TGACGACCAAGGTTCCATGT-3′; firefly luciferase: forward 5′-ATCCGGAAGCGACCAACGCC-3′, reverse 5′-GTCGGGAAGACCTGCCACGC-3′.

### 2.9 RT-qPCR on total RNA

Extracted total RNA from the Ribosome Profiling samples was used for qPCR. 1 μg of each sample was reverse transcribed into cDNA using SuperScript™ IV VILO™ Master Mix (ThermoFisher Scientific). Appropriate dilutions of the cDNA were used in the qPCR reaction, using PowerUp™ SYBR™ Green Master Mix (Thermofisher). Primers were used at 5 μM and cycling conditions were according to the manufacturer’s specifications. Reactions were run in an AriaMx Real-time PCR System. Raw data were analysed using the AriaMx software. Expression fold change was calculated using the ΔΔC_t_ method, normalising to loading control and home cage.

$$FC=\;2^{\left(\left(C_{t,GIE}-C_{t,LC}\right)-\left(C_{t,GIC}-C_{t,LC}\right)\right)}$$

$C_{t}$ is the cycle threshold (number of cycles at which the signal exceeds background); $C_{t,GIE}$ is the value for the gene of interest in the experimental condition, $C_{t,GIC}$ the value for the control in the experimental condition, and $C_{t,LC}$ the value for the loading control (housekeeping gene). The following primers were used: *Egr2*: forward 5′- CACCTAGAAACCAGACCTTCAC-3′, reverse 5′- GATGCCCGCACTCACAATA-3′; *Cfos*: forward 5′-ATTGTCGAGGTGGTCTGAATG-3′, reverse 5′-TCGAAAGACCTCAGGGTAGAA-3′; *Arc*: forward 5′-GGAGGGAGGTCTTCTACCGTC-3′, reverse 5′−CCCCCACACCTACAGAGACA-3′; *Coll11a1*: forward 5′-GGCTGAGAGTGTAACAGAGAT-3′, reverse 5′-TAGGAGTCTCAGTCTGGTAAGG-3′; *Robo3*: forward 5′- CTTAAGGAAGAGGAGGGAAGGA-3′, reverse 5′-GTTGGAGGCTACGCACATATAC-3′; *Leng8*: forward 5′-GGGTTCCAGATACTTGGTAAGG-3′, reverse 5′-AGTGCCTTCTGGTTGTTACTC-3′;

### 2.10 Immunoblotting

Various tissues (hippocampus, kidney, liver, muscle or spleen) were rapidly isolated from C57BL/6 mice, age 8 weeks, and lysed in RIPA buffer (150 mM NaCl, 1.0 % NP-40, 0.5 % sodium deoxycholate, 0.1 % SDS, 50 mM Tris, pH 8.0) supplemented with protease and phosphatase inhibitors (Roche), using a Dounce glass homogeniser by applying ∼30 strokes, on ice. Samples were further incubated on ice for 15 min, with occasional vortexing, and cleared by centrifugation for 20 min at 16,000 x *g* at 4 °C. Protein concentration of each sample was determined by measuring A_280_ absorbance of the supernatant on a NanoDrop (ThermoFisher Scientific). 50 μg of protein per lane was prepared in Laemmli sample buffer (50 mM Tris, pH 6.8, 100 mM DTT, 2 % SDS, 10 % glycerol, 0.1 % bromophenol blue), heated to 95 °C for 2 min, and resolved on 10 %–16 % polyacrylamide gels. Proteins were transferred to a 0.2 μm nitrocellulose membrane (Bio-Rad), blocked in 5 % BSA in TBS-T (10 mM Tris, pH 7.6, 150 mM NaCl, 0.1 % Tween20) for 45 min at room temperature, incubated with primary antibodies 1:1000 (1 % BSA in TBS-T containing 0.02 % Na azide) overnight at 4 °C and with secondary antibodies 1:5000 for 1 h at room temperature (1 % BSA in TBS-T containing 0.02 % Na azide). Between incubations, membranes were washed three times in TBS-T. For reprobing, membranes were stripped by incubation with 0.2 M NaOH for 5 min and blocked with 5 % BSA in TBS-T for 1 h. Blots were imaged using an Odyssey Imaging System (Li−COR Biosciences) at a resolution of 169 μm. Primary antibodies used: Ribosomal Protein S6 Antibody (C-8); sc-74459, Santa Cruz Biotechnology, Ribosomal Protein L13a Antibody; 2765, Cell Signalling Technologies, Ribosomal Protein S15 Antibody; ab157193, abcam, Ribosomal Protein L11 (D1P5N); 18163S, Cell Signalling Technologies, Ribosomal Protein L10a; ab174318, abcam and Hsc-70; sc-7298, Santa Cruz Biotechnologies).

### 2.11 Statistical analysis

Experimenters were blinded to the group identity during data analysis. All data are presented as mean ± S.E.M. (error bars) and individual experimental points are depicted in column or bar graphs. Statistical significance was set *a priori* at 0.05 (n.s.: non-significant). Where analysis of variance (ANOVA) was carried out the assumptions for normality (Shapiro-Wilk) and equality of variances (Bartlett’s test) were met. No nested data were obtained in this study; we only collected one observation per research object. The n number denotes biological replicates. No randomization was carried out for any of the experiments described here. Details for statistical and post-hoc tests (p-value, F-ratio) used were provided within figure legends or the relative methods description and summarised in Supplementary Table 5; all data collected followed normal distributions, thus only parametric tests were used. Data summaries and statistical analysis were carried out using Graphpad Prism 6 and or SPSS version 20 unless otherwise stated.

## Data availability

All sequencing and pathway analysis data is deposited in Mendeley:

DOI: https://doi.org/10.17632/8hrj49fthr.2.

## CRediT authorship contribution statement

**Konstanze Simbriger:** Methodology, Investigation, Writing - review & editing. **Inês S. Amorim:** Investigation, Methodology, Writing - review & editing. **Gilliard Lach:** Investigation, Methodology, Writing - review & editing. **Kleanthi Chalkiadaki:** Investigation, Methodology, Writing - review & editing. **Stella Kouloulia:** Investigation, Methodology, Writing - review & editing. **Seyed Mehdi Jafarnejad:** Conceptualization, Methodology, Investigation, Writing - review & editing, Supervision. **Arkady Khoutorsky:** Conceptualization, Methodology, Investigation, Writing - original draft, Writing - review & editing, Supervision. **Christos G. Gkogkas:** Conceptualization, Methodology, Investigation, Writing - original draft, Writing - review & editing, Funding acquisition, Supervision.

## Declaration of Competing Interest

The authors report no declarations of interest.
