# Supplementary material for: Uncovering memory-related gene expression in contextual fear conditioning using ribosome profiling
Source: Prog Neurobiol. 2021 Feb;197:101903. doi: 10.1016/j.pneurobio.2020.101903 (PMC7859833; doi:10.1016/j.pneurobio.2020.101903)
Supplement: Supplementary file 2 [file mmc2.docx]

Supplementary Information

**Supplementary Figure 1. Polysome profiling in brain/neurons/cells and analysis of the translational efficiency of mRNAs encoding ribosomal proteins in different tissues and cell types.** **a.** Polysome profiling analysis of the indicated groups. Continuous UV absorbance at 254 nm of lysates resolved over a 5-50% sucrose gradient. 40S, 60S, 80S (monosome), light and heavy polysomes are marked on the absorbance graph. **b.** Immunoblot of lysates prepared from the indicated tissues probed with antisera against the indicated large and small ribosomal proteins; Hsc70 is a loading control. **c.** Box plots of ribosome profiling data (log_10_ of translational efficiency; TE) for mRNAs that encode all proteins, ribosomal proteins, or mitochondrial ribosomal proteins from the following studies: this paper: Dissociated DIV25 mouse cortical neurons, dorsal hippocampus from the homecage group, mouse embryonic stem cells (mESC)^35^, Kidney^36^, Liver^37^, Muscle^38^ and from ref.^5^ (Control CFC, Dissociated Neurons), all summarised in Supplementary Table 4. ***p<0.001, **p<0.01, *p<0.05; One-way ANOVA with Bonferroni’s post-hoc.

**Supplementary Figure 2. Quality control and validation of the ribosome profiling assay. a.** Frequency versus length of mapped reads for total mRNA and footprint libraries **b.** Cumulative reading frame usage for total mRNA and footprint libraries. **c.** Frequency of reads for footprint (black; showing the expected 3 nt periodicity) and total mRNA libraries (grey) in relation to 5′ read position (start and stop codon shown). **d.** Reproducibility plots (n=2) for sequenced libraries [for replicates of total mRNA and footprints (light grey corresponds to data points with <40 reads)]; see also Materials and Methods. **e.** Correlation matrices for the RPKM and TE of all replicates and all experimental groups, demonstrating the replicate and condition changes.

All data are shown for the three experimental groups (homecage, shock only, CFC).

**Supplementary Figure 3. Principal Components Analysis of RNA sequencing. a.** Translational efficiency and **b.** Transcription biological replicates for all groups; PC: principal component.

**Supplementary Figure 4. Comparison of translation and transcription of top genes from Fig. 1 within biological replicates.** Log_2_ expression (fold change) between biological replicates for immediate shock and CFC groups presented for the indicated genes from Fig. 1; n=2 (see methods), Student’s *t-*test. Data are shown as mean ±S.E.M.
