# Supplementary material for: Uncovering memory-related gene expression in contextual fear conditioning using ribosome profiling
Source: Prog Neurobiol. 2021 Feb;197:101903. doi: 10.1016/j.pneurobio.2020.101903 (PMC7859833; doi:10.1016/j.pneurobio.2020.101903)
Supplement: Supplementary file 3 [file mmc3.docx]

**Supplementary Table 1** Review of CFC gene expression literature

**Supplementary Table 2** List of DTGs and DEGs from Ribosome Profiling

**Supplementary Table 3** IPA and DAVID analysis of DTGs and DEGs (bundle) -

**Supplementary Table 4** RNA sequencing studies used in this paper

**Supplementary Table 5** Statistical Analysis
