# Supplementary material for: Uncovering memory-related gene expression in contextual fear conditioning using ribosome profiling
Source: Prog Neurobiol. 2021 Feb;197:101903. doi: 10.1016/j.pneurobio.2020.101903 (PMC7859833; doi:10.1016/j.pneurobio.2020.101903)
Supplement: Supplementary file 4 [file mmc4.pdf]

Supplementary Table

Reference	Test	Control group	Gene expression
1	AFC	NS/HC	Genome-wide
2	CFC	NS	Specific-mir138
3	AFC	SO/TO	Specific-Fos
4	AFC	TO/SO	Genome wide
5	CFC	HC	Genome-wide
6	AFC	US	Genome-wide
7	CFC	HC	Specific-numerous
8	CFC	SO	Genome-wide
9	CFC	NS	Genome-wide
10	CFC	NI	Specific-Syt13
11	CFC	HC	Genome-wide
12	AFC	HC	Genome-wide
13	AFC/CFC	NI	Genome-wide
14	CFC	SO/NS	Genome-wide
15	TFC	HC/SO/TO	Genome-wide
16	AFC	HC/SO/TO/NS	Genome-wide
17	CFC	SO/NS	Specific-c-fos/ NGFI-A

NS- No Shock (chamber exposure, no footshock)

HC-home cage

SO-shock only

TO-Tone only

US-Unpaired shock

NI-Not indicated

CFC-contextual fear conditioning, AFC-auditory/cued fear conditioning, TFC-trace fear conditioning.

1 Rao-Ruiz, P. *et al.* Engram-specific transcriptome profiling of contextual memory consolidation. *Nature communications* **10**, 2232, doi:10.1038/s41467-019-09960-x (2019).

2 Li, D. W. *et al.* Fear conditioning downregulates miR-138 expression in the hippocampus to facilitate the formation of fear memory. *Neuroreport* **29**, 1418-1424, doi:10.1097/WNR.0000000000001129 (2018).

3 Cho, J. H., Rendall, S. D. & Gray, J. M. Brain-wide maps of Fos expression during fear learning and recall. *Learn Mem* **24**, 169-181, doi:10.1101/lm.044446.116 (2017).

4 Cho, J. H., Huang, B. S. & Gray, J. M. RNA sequencing from neural ensembles activated during fear conditioning in the mouse temporal association cortex. *Scientific reports* **6**, 31753, doi:10.1038/srep31753 (2016).

5 Cho, J. *et al.* Multiple repressive mechanisms in the hippocampus during memory formation. *Science* **350**, 82-87, doi:10.1126/science.aac7368 (2015).

6 Katz, I. K. & Lamprecht, R. Fear conditioning leads to alteration in specific genes expression in cortical and thalamic neurons that project to the lateral amygdala. *Journal of neurochemistry* **132**, 313-326, doi:10.1111/jnc.12983 (2015).

7 Poplawski, S. G. *et al.* Object-location training elicits an overlapping but temporally distinct transcriptional profile from contextual fear conditioning. *Neurobiology of learning and memory* **116**, 90-95, doi:10.1016/j.nlm.2014.09.002 (2014).
8 Federighi, G. *et al.* Modulation of gene expression in contextual fear conditioning in the rat. *PloS one* **8**, e80037, doi:10.1371/journal.pone.0080037 (2013).
9 Park, C. S., Rehrauer, H. & Mansuy, I. M. Genome-wide analysis of H4K5 acetylation associated with fear memory in mice. *BMC Genomics* **14**, 539, doi:10.1186/1471-2164-14-539 (2013).
10 Han, S. *et al.* Altered expression of synaptotagmin 13 mRNA in adult mouse brain after contextual fear conditioning. *Biochemical and biophysical research communications* **425**, 880-885, doi:10.1016/j.bbrc.2012.07.166 (2012).
11 Ainsley, J. A., Drane, L., Jacobs, J., Kittelberger, K. A. & Reijmers, L. G. Functionally diverse dendritic mRNAs rapidly associate with ribosomes following a novel experience. *Nature communications* **5**, 4510, doi:10.1038/ncomms5510 (2014).
12 Eacker, S. M. *et al.*, doi:10.1101/169425 (2017).
13 Mei, B. *et al.* Distinct gene expression profiles in hippocampus and amygdala after fear conditioning. *Brain Res Bull* **67**, 1-12, doi:10.1016/j.brainresbull.2005.03.023 (2005).
14 Bero, A. W. *et al.* Early remodeling of the neocortex upon episodic memory encoding. *Proceedings of the National Academy of Sciences of the United States of America* **111**, 11852-11857, doi:10.1073/pnas.1408378111 (2014).
15 Sirri, A. *et al.* Temporal gene expression profile of the hippocampus following trace fear conditioning. *Brain research* **1308**, 14-23, doi:10.1016/j.brainres.2009.10.049 (2010).
16 Ploski, J. E., Park, K. W., Ping, J., Monsey, M. S. & Schafe, G. E. Identification of plasticity-associated genes regulated by Pavlovian fear conditioning in the lateral amygdala. *Journal of neurochemistry* **112**, 636-650, doi:10.1111/j.1471-4159.2009.06491.x (2010).
17 Rosen, J. B., Fanselow, M. S., Young, S. L., Sitcoske, M. & Maren, S. Immediate-early gene expression in the amygdala following footshock stress and contextual fear conditioning. *Brain research* **796**, 132-142, doi:10.1016/s0006-8993(98)00294-7 (1998).
