# Supplementary material for: Uncovering memory-related gene expression in contextual fear conditioning using ribosome profiling
Source: Prog Neurobiol. 2021 Feb;197:101903. doi: 10.1016/j.pneurobio.2020.101903 (PMC7859833; doi:10.1016/j.pneurobio.2020.101903)
Supplement: Supplementary file 7 [file mmc7.pdf]

Supplementary Table 4 Statistical Analysis

Test	**Mean ± S.E.M.**	**Significance and multiple comparisons**	**Parameter**	**N**	**Descriptive Statistics**	**Figure**
One-way ANOVA; Tukey's post-hoc	homecage:<br>7.517 ± 0.802<br><br>shock only:<br>8.150 ± 1.385<br><br>CFC:<br>68.580 ± 1.731	homecage vs. shock only p=0.9422<br>homecage vs. CFC p<0.0001<br>shock only vs. CFC p<0.0001	%Freezing	All groups (12)	F (2, 33) = 663.9<br>p<0.0001	Fig. 1a
One-way ANOVA; Bonferonni's post-hoc	**CFC-shock**<br>Length 5′ UTR<br>DTG up:<br>192.600 ± 26.360<br><br>DTG down:<br>366.000 ± 87.46<br><br>GC (%) 5′ UTR<br>DTG up:<br>60.250 ± 1.599<br><br>DTG down:<br>60.490 ± 1.651<br><br>Gibbs Free Energy (kcal/mol) 5′ UTR<br>DTG up:<br>-73.230 ± 12.17<br><br>DTG down:<br>-135.7 ± 26.74<br><br>Length 3′ UTR<br>DTG up:<br>1737.000 ± 334.100<br>DTG down:<br>1471 ± 225.300<br><br>GC (%) 3′ UTR<br>DTG up:<br>43.410 ±1.217<br>DTG down:<br>49.010 ± 1.392<br><br>Gibbs Free Energy (kcal/mol) 3′ UTR<br>DTG up:<br>-458.600 ± 76.88<br>DTG down:<br>-493.8 ± 77.61<br><br>**shock - CFC**<br>Length 5′ UTR<br>DTG up:<br>193.800 ± 23.030<br>DTG down: 306.000 ± 54.670	**CFC-shock**<br>Length 5′ UTR DTG up vs DTG down p=0.401<br>GC (%)5′ UTR DTG up vs DTG down p>0.999<br>Gibbs Free Energy (kcal/mol) 5′ UTR DTG up vs DTG down p=0.017<br><br>Length 3′ UTR DTG up vs DTG down p>0.999<br>GC (%) 3′ UTR DTG up vs DTG down p=0.0166<br>Gibbs Free Energy (kcal/mol) 3′ UTR DTG up vs DTG down p>0.999<br><br>**shock-CFC**<br>Length 5′ UTR DTG up vs DTG down p=0.401<br>GC (%)5′ UTR DTG up vs DTG down p>0.999<br>Gibbs Free Energy (kcal/mol) 5′ UTR DTG up vs DTG down p=0.273<br><br>Length 3′ UTR DTG up vs DTG down p<0.0001<br>GC (%) 3′ UTR DTG up vs DTG down p=0.02<br>Gibbs Free Energy (kcal/mol) 3′ UTR DTG up vs DTG down p<0.0001	Length of UTR (bp), or Percentage GC, or Gibbs Free Energy (kcal/mol)	**CFC-shock**<br>DTG up (38), DTG down (31)<br>**shock-CFC**<br>DTG up (67), DTG down (76)	Length 5′ UTR<br>F (3, 208) = 2.461<br>p=0.063<br><br>GC (%)5′ UTR<br>F (3, 208) = 0.6619<br>p=0.576<br><br>Gibbs Free Energy (kcal/mol) 5′ UTR<br>F (3, 207) = 2.878<br>p=0.037<br><br>Length 3′ UTR<br>F (3, 208) = 11.58<br>p<0.0001<br><br>GC (%)3′ UTR<br>p=0.0005<br><br>Gibbs Free Energy (kcal/mol) 3′ UTR<br>p<0.0001	Fig. 3a

	GC (%)5′ UTR<br>DTG up:<br>61.73 ± 1.077<br>DTG down: 63.15 ± 1.487<br><br>Gibbs Free Energy (kcal/mol) 5′ UTR<br>DTG up: -73.410 ± 9.073<br>DTG down: -114.700 ± 18.040<br><br>Length 3′ UTR<br>DTG up: 665.400 ± 84.680<br>DTG down: 2156 ± 210.4<br><br>GC (%)3′ UTR<br>DTG up: 46.800 ± 0.968<br>DTG down: 43.11 ± 0.846<br><br>Gibbs Free Energy (kcal/mol) 3′ UTR<br>DTG up: -211.200 ± 27.390<br>DTG down: -601.3 ± 57.100					
Two-way ANOVA; Bonferonni's post-hoc	**CFC UP**<br>5′ UTR<br>IRES<br>21.052630<br>PG4 7.894737<br>TOP 0.000000<br>uORF<br>26.315790<br><br>3′ UTR<br>BRD-BOX<br>13.157900<br>CPE 10.526320<br>GRE 18.421050<br>GY-BOX 7.894737<br>K-BOX 28.947370<br>PAS 81.578950<br>PG4 18.421050<br>SXL_BS<br>18.421050<br>UNR_BS<br>21.052630<br><br>**CFC DOWN**<br>5′ UTR	shock only UP vs. shock only DOWN p>0.999<br>shock only UP vs. CFC unique UP p>0.999<br>shock only UP vs. CFC unique DOWNp<0.0001<br>shock only DOWN vs. CFC unique UP p>0.999<br>shock only DOWN vs. CFC unique DOWN p<0.0001<br>CFC unique UP vs. CFC unique DOWN p<0.0001<br>PG4 (all p>0.999)<br>shock only UP vs. shock only DOWN<br>shock only UP vs. CFC unique UP<br>shock only UP vs. CFC unique DOWN	Motif for UTRdb per experimental group (5′ or 3′ UTR) per upregulated or downregulated DTGs	uORF<br><br>N in figure legend	5′ UTR<br>Motif effect: F (3, 9) = 16.37<br>p=0.0005<br>group effect:<br>F (3, 9) = 1.373<br>p=0.312<br><br>3′ UTR<br>Motif effect: F (8, 24) = 102.8 p<0.0001<br>group effect:<br>F (3, 24) = 9.376,<br>p>0.999	Fig. 3b

	IRES 25.806450<br>PG4 6.451613<br>TOP 0.000000<br>uORF 54.838710<br><br>3′ UTR<br>BRD-BOX 3.225807<br>CPE 3.225807<br>GRE 9.677420<br>GY-BOX 9.677420<br>K-BOX 12.903230<br>PAS 90.322580<br>PG4 6.451613<br>SXL_BS 12.903230<br>UNR_BS 19.354840<br><br>**Shock UP**<br>5′ UTR<br>IRES 17.910450<br>PG4 2.985075<br>TOP 4.477612<br>uORF 23.880600<br>3′ UTR<br>BRD-BOX 5.97014<br>CPE 4.477612<br>GRE 1.492537<br>GY-BOX 5.97014<br>K-BOX 8.955224<br>PAS 85.074620<br>PG4 5.970149<br>SXL_BS 2.98507<br>UNR_BS 4.47761<br><br>**Shock DOWN**<br>5′ UTR<br>IRES 25.000000<br>PG4 10.526320<br>TOP 3.947368<br>uORF 31.578950<br><br>3′ UTR<br>BRD-BOX<br>17.105260<br>CPE 3.947368<br>GRE 15.789470<br>GY-BOX 22.368420<br>K-BOX 28.947370<br>PAS 92.105260<br>PG4 7.894737<br>SXL_BS 15.789470<br>UNR_BS 18.421050	shock only DOWN vs. CFC unique UP<br>shock only DOWN vs. CFC unique DOWN<br>CFC unique UP vs. CFC unique DOWN<br>TOP (all p>0.999)<br>shock only UP vs. shock only DOWN<br>shock only UP vs. CFC unique UP<br>shock only UP vs. CFC unique DOWN<br>shock only DOWN vs. CFC unique UP<br>shock only DOWN vs. CFC unique DOWN<br>CFC unique UP vs. CFC unique DOWN<br>IRES (all p>0.999)<br>shock only UP vs. shock only DOWN<br>shock only UP vs. CFC unique UP<br>shock only UP vs. CFC unique DOWN<br>shock only DOWN vs. CFC unique UP<br>shock only DOWN vs. CFC unique DOWN<br>CFC unique UP vs. CFC unique DOWN<br><br>BRD-BOX<br>shock only UP vs. shock only DOWN p=0.775<br>shock only UP vs. CFC unique UP p>0.999<br>shock only UP vs. CFC unique DOWN p>0.999<br>shock only DOWN vs. CFC unique UP p>0.999<br>shock only DOWN vs. CFC unique DOWN p=0.371<br>CFC unique UP vs. CFC unique DOWN p>0.999<br>GRE<br>shock only UP vs. shock only DOWN p=0.329<br>shock only UP vs. CFC unique UP p=0.150<br>shock only UP vs. CFC unique DOWN p>0.999<br>shock only DOWN vs. CFC unique UP p>0.999<br>shock only DOWN vs. CFC unique DOWN p>0.999				

		CFC unique UP vs. CFC unique DOWN p>0.999<br>CPE (all p>0.999)<br>shock only UP vs. shock only DOWN<br>shock only UP vs. CFC unique UP<br>shock only UP vs. CFC unique DOWN<br>shock only DOWN vs. CFC unique UP<br>shock only DOWN vs. CFC unique DOWN<br>CFC unique UP vs. CFC unique DOWN<br>GY-BOX<br>shock only UP vs. shock only DOWN p=0.177<br>shock only UP vs. CFC unique UP p>0.999<br>shock only UP vs. CFC unique DOWN p>0.999<br>shock only DOWN vs. CFC unique UP p=0.313<br>shock only DOWN vs. CFC unique DOWN p=0.515<br>CFC unique UP vs. CFC unique DOWN p>0.999<br>K-BOX<br>shock only UP vs. shock only DOWN p=0.056<br>shock only UP vs. CFC unique UP p=0.056<br>shock only UP vs. CFC unique DOWN p>0.999<br>shock only DOWN vs. CFC unique UP p>0.999<br>shock only DOWN vs. CFC unique DOWN p=0.197<br>CFC unique UP vs. CFC unique DOWN p=0.197<br>PAS<br>shock only UP vs. shock only DOWN p>0.999<br>shock only UP vs. CFC unique UP p>0.999<br>shock only UP vs. CFC unique DOWN p>0.999<br>shock only DOWN vs. CFC unique UP p= 0.9029<br>shock only DOWN vs. CFC unique DOWN pp>0.999				

		CFC unique UP vs. CFC unique DOWN p=0.625<br>PG4<br>shock only UP vs. shock only DOWN<br>shock only UP vs. CFC unique UP<br>shock only UP vs. CFC unique DOWN<br>shock only DOWN vs. CFC unique UP<br>shock only DOWN vs. CFC unique DOWN<br>CFC unique UP vs. CFC unique DOWN<br>SXL_BS<br>shock only UP vs. shock only DOWN p=0.500<br>shock only UP vs. CFC unique UP p=0.236<br>shock only UP vs. CFC unique DOWN p>0.999<br>shock only DOWN vs. CFC unique UP p>0.999<br>shock only DOWN vs. CFC unique DOWN p>0.999<br>CFC unique UP vs. CFC unique DOWN p>0.999<br>UNR_BS<br>shock only UP vs. shock only DOWN p=0.364<br>shock only UP vs. CFC unique UP p=0.168<br>shock only UP vs. CFC unique DOWN p=0.279<br>shock only DOWN vs. CFC unique UP p>0.999<br>shock only DOWN vs. CFC unique DOWN p>0.999<br>CFC unique UP vs. CFC unique DOWN p>0.999				
One-way ANOVA; Tukey's posthoc	Homecage: 5.033 ± 0.550<br>Shock: 5.003 ± 0.103<br>CFC: 5.006 ± 0.080	homecage vs shock p=0.963<br>homecage vs CFC p=0.969<br>shock vs CFC p=0.999	Polysome/monosome ratio	N=4 for each group	$F_{(2, 9)} = 0.04194$<br>$p=0.959$	Fig. 4a
One-way ANOVA; Tukey's posthoc	**Sumo1**<br>Homecage 1.339±0.084<br>Shock 2.279±0.140<br>CFC 2.436±0.196<br><br>**Rpl37**	**Sumo1**<br>Homecage vs Shock p=0.0038<br>Homecage vs CFC p=0.00014<br>Shock vs CFC p=0.7401	Ratio mRNA abundance heavy/light polysomes	N=4 for each group	**Sumo1**<br>$F_{(2, 9)} = 16.16$<br>$P=0.0017$<br><br>**Rpl37**<br>$F_{(2, 9)} = 27.81$<br>$p=0.0001$	Fig. 4b

	Homecage 1.333±0.110<br>Shock 2.447±0.112<br>CFC 2.591±0.162<br><br>**Npas4**<br>Homecage 1.406±0.068<br>Shock 2.645±0.093<br>CFC 2.544±0.030<br><br>**Rpl27**<br>Homecage 1.549±0.029<br>Shock 1.465±0.073<br>CFC 2.191±0.100<br><br>**Xkr8**<br>Homecage 1.535±0.087<br>Shock 1.577±0.033<br>CFC 2.390±0.134<br><br>**Tfb2m**<br>Homecage 1.359±0.079<br>Shock 1.500±0.133<br>CFC 2.503±0.145	**Rpl37**<br>Homecage vs Shock p=0.0005<br>Homecage vs CFC p=0.0005<br>Shock vs CFC p=0.724<br><br>**Npas4**<br>Homecage vs Shock p<0.0001<br>Homecage vs CFC p<0.0001<br>Shock vs CFC p=0.576<br><br>**Rpl27**<br>Homecage vs Shock p=0.711<br>Homecage vs CFC p=0.0004<br>Shock vs CFC p=0.0002<br><br>**Xkr8**<br>Homecage vs Shock p=0.946<br>Homecage vs CFC p=0.0003<br>Shock vs CFC p=0.0005<br><br>**Tfb2m**<br>Homecage vs Shock p=0.703<br>Homecage vs CFC p=0.0003<br>Shock vs CFC p=0.0007			**Npas4**<br>F (2, 9) = 98.92<br>p<0.0001<br><br>**Rpl27**<br>F (2, 9) = 28.91<br>p=0.0001<br><br>**Xkr8**<br>F (2, 9) = 25.97<br>p=0.0002<br><br>**Tfb2m**<br>F (2, 9) = 25.79<br>p=0.0002	
One-way ANOVA; Tukey's posthoc	**Fos**<br>Homecage 1.007 ±0.116<br>Shock .761±0.061<br>CFC 4.462±0.061<br><br>**Egr2**<br>Homecage 1.000±0.020<br>Shock 3.204±0.541<br>CFC 3.974±0.203<br><br>**Arc**<br>Homecage 1.006±0.107<br>Shock 1.949±0.119<br>CFC 2.402±0.074<br><br>**Col11a1**<br>Homecage 1.003±0.071	**Fos**<br>Homecage vs Shock=<0.0001<br>Homecage vs CFC p=0.0001<br>Shock vs CFC p=0.165<br><br>**Egr2**<br>Homecage vs Shock p=0.037<br>Homecage vs CFC p=0.016<br>Shock vs CFC p=0.360<br><br>**Arc**<br>Homecage vs Shock p=0.014<br>Homecage vs CFC p=0.004	Log_2 (Expression Fold Change)	N=2 for each group	**Fos**<br>F (2, 3) = 616.3<br>p=0.0001<br><br>**Egr2**<br>F (2, 3) = 21.34<br>p=0.0168<br><br>**Arc**<br>F (2, 3) = 48.46<br>p=0.0050<br><br>**Col11a1**<br>F (2, 3) = 178.4<br>P=0.0008<br><br>**Robo3**<br>F (2, 3) = 21.60<br>P=0.0165<br><br>**Leng8**	Fig. 4c

	Shock 0.587±0.170<br>CFC 3.355±0.058<br><br>**Robo3**<br>Homecage<br>1.000±0.013<br>Shock 1.013±0.157<br>CFC 4.424±0.718<br><br>**Leng8**<br>Homecage<br>1.001±0.050<br>Shock 0.749±0.013<br>CFC 3.483±0.159	Shock vs CFC<br>p=0.102<br><br>**Col11a1**<br>Homecage vs Shock<br>p=0.151<br>Homecage vs CFC<br>p=0.001<br>Shock vs CFC<br>p=0.0009<br><br>**Robo3**<br>Homecage vs Shock<br>p=0.999<br>Homecage vs CFC<br>p=0.021<br>Shock vs CFC<br>p=0.021<br><br>**Leng8**<br>Homecage vs Shock<br>p=0.300<br>Homecage vs CFC<br>p=0.0008<br>Shock vs CFC<br>p=0.0008			F (2, 3) = 242.3<br>p=0.0005	
One-way ANOVA; Bonferonni's posthoc	**HEK**<br>All proteins<br>0.175±0.005<br><br>Ribosome -<br>0.104±0.056<br><br>Mitochondrial<br>Ribosome<br>0.209±0.084<br><br>**Diss. Neurons**<br>All proteins -<br>0.064±0.002<br><br>Ribosome -<br>0.597±0.056<br><br>Mitochondrial<br>Ribosome -<br>0.035±0.028<br><br>**Dorsal Hipp. Homecage**<br>All proteins -<br>0.052±0.002<br><br>Ribosome -<br>0.612±0.059<br><br>Mitochondrial<br>Ribosome -<br>0.112±0.027<br><br>**mESC (Ingolia et al. 2011)**	**HEK**<br>All proteins vs<br>Ribosome p<0.0001<br><br>All proteins vs<br>Mitochondrial<br>Ribosome p>0.999<br><br>Ribosome vs<br>Mitochondrial<br>Ribosome p=0.011<br><br>**Diss. Neurons**<br>All proteins vs<br>Ribosome p<0.0001<br><br>All proteins vs<br>Mitochondrial<br>Ribosome p>0.999<br><br>Ribosome vs<br>Mitochondrial<br>Ribosome p<0.0001<br><br>**Dorsal Hipp. Homecage**<br>All proteins vs<br>Ribosome p<0.0001<br><br>All proteins vs<br>Mitochondrial<br>Ribosome p=0.392<br><br>Ribosome vs<br>Mitochondrial<br>Ribosome p<0.0001	Log_{10} (Translational Efficiency)	**HEK**<br>(13846, 77, 57)<br>**Diss. Neurons**<br>(15685, 75, 74)<br>**Dorsal Hipp. Homecage**<br>(15963, 75, 75)<br>**mESC (Ingolia et al. 2011)**<br>(10074, 78, 78)<br>**Kidney**<br>(17600, 78, 78)<br>**Liver**<br>(14797, 76, 78)<br>**Muscle**<br>(18122, 78, 78)<br>**Cho et al. CFC**<br>(14267, 64, 78)<br>**Cho et al. Diss. Neurons**<br>(11891, 52, 77)	**HEK**<br>F (2, 13977) = 7.910<br>p=0.0004<br>**Diss. Neurons**<br>F (2, 15831) = 71.42<br>p<0.0001<br>**Dorsal Hipp. Homecage**<br>F (2, 16110) = 105.1<br>p<0.0001<br>**mESC (Ingolia et al. 2011)**<br>F (2, 10225) = 33.92<br>p<0.0001<br>**Kidney**<br>F (2, 17753) = 72.50<br>p<0.0001<br>**Liver**<br>F (2, 14948) = 152.0<br>p<0.0001<br>**Muscle**<br>F (2, 18273) = 49.85<br>p<0.0001<br>**Cho et al. CFC**<br>F (2, 14406) = 67.21<br>p<0.0001<br>**Cho et al. Diss. Neurons**<br>F (2, 12017) = 33.09<br>p<0.0001	Sup. Fig. 1c

	All proteins<br>0.109±0.002<br><br>Ribosome -<br>0.121±0.02<br><br>Mitochondrial<br>Ribosome<br>0.172±0.013<br><br>**Kidney**<br>All proteins -<br>0.165±0.003<br><br>Ribosome -<br>0.719±0.050<br><br>Mitochondrial<br>Ribosome<br>0.053±0.027<br><br>**Liver**<br>All proteins -<br>0.266±0.003<br><br>Ribosome -<br>0.965±0.063<br><br>Mitochondrial<br>Ribosome -<br>0.027±0.023<br><br>**Muscle**<br>All proteins -<br>0.169±0.004<br><br>Ribosome -<br>0.785±0.051<br><br>Mitochondrial<br>Ribosome 0.019±-<br>0.027<br><br>**Cho et al. CFC**<br>All proteins -<br>0.052±0.003<br><br>Ribosome -<br>0.597±0.087<br><br>Mitochondrial<br>Ribosome<br>0.110±0.035<br><br>**Cho et al. Diss. Neurons**<br>All proteins -<br>0.073±0.003<br><br>Ribosome -<br>0.495±0.099	**mESC (Ingolia et al. 2011)**<br>All proteins vs<br>Ribosome p=0.0009<br><br>All proteins vs<br>Mitochondrial<br>Ribosome p>0.999<br><br>Ribosome vs<br>Mitochondrial<br>Ribosome p=0.0082<br><br>**Kidney**<br>All proteins vs<br>Ribosome p<0.0001<br><br>All proteins vs<br>Mitochondrial<br>Ribosome p=0.0002<br><br>Ribosome vs<br>Mitochondrial<br>Ribosome p<0.0001<br><br>**Liver**<br>All proteins vs<br>Ribosome p<0.0001<br><br>All proteins vs<br>Mitochondrial<br>Ribosome p=0.0004<br><br>Ribosome vs<br>Mitochondrial<br>Ribosome p<0.0001<br><br>**Muscle**<br>All proteins vs<br>Ribosome p<0.0001<br><br>All proteins vs<br>Mitochondrial<br>Ribosome p<0.0001<br><br>Ribosome vs<br>Mitochondrial<br>Ribosome p<0.0001<br><br>**Cho et al. CFC**<br>All proteins vs<br>Ribosomep<0.0001<br><br>All proteins vs<br>Mitochondrial<br>Ribosome p=0.356<br><br>Ribosome vs<br>Mitochondrial<br>Ribosome p<0.001				

	Mitochondrial Ribosome - 0.002±0.040	**Cho et al. Diss. Neurons**<br>All proteins vs Ribosome p<0.0001<br><br>All proteins vs Mitochondrial Ribosome p>0.999<br><br>Ribosome vs Mitochondrial Ribosome p<0.0001				
