# Supplementary material for: Uncovering memory-related gene expression in contextual fear conditioning using ribosome profiling
Source: Prog Neurobiol. 2021 Feb;197:101903. doi: 10.1016/j.pneurobio.2020.101903 (PMC7859833; doi:10.1016/j.pneurobio.2020.101903)
Supplement: Supplementary file 1 [file mmc1.pdf]

# Peer Review Overview

**Manuscript Title: “Uncovering memory-related gene expression in contextual fear conditioning using ribosome profiling”**



| | |
|---|---|
| Received | 29-Oct-2019 |
| 1st Decision | 26-Dec-2019 |
| 1st Revision Submitted | 19-Feb-2020 |
| 2nd Decision | 05-May-2020 |
| 2nd Revision Submitted | 30-Jun-2020 |
| Accepted | 23-Aug-2020 |



---

## 1st Decision Letter

Dear Dr. Gkogkas,

Thank you for submitting your manuscript to Progress in Neurobiology.

We have completed our evaluation of your manuscript. The reviewers recommend reconsideration of your manuscript following major revision. We invite you to resubmit your manuscript after addressing the comments below. Please resubmit your revised manuscript by Feb 24, 2020.

When revising your manuscript, please consider all issues mentioned in the reviewers' comments carefully: please outline every change made in response to their comments and provide suitable rebuttals for any comments not addressed. Please note that your revised submission may need to be re-reviewed.

Progress in Neurobiology values your contribution and we look forward to receiving your revised manuscript.

Kind regards,

Laura Colgin
Associate Editor

Sabine Kastner
Editor-in-Chief
Progress in Neurobiology

**Editor and Reviewer comments:**

**Reviewer 1**

The current manuscript "Uncovering salient memory genes in contextual fear conditioning using ribosome profiling" by Drs. Sibriger and colleagues aims to distinguish between gene expression as a consequence of context fear memory formation, and gene expression as a

consequence of footshock "alone". Importantly - and perhaps not surprisingly - they find that a small number of genes, and in particular, immediate early genes, show increased expression in dorsal hippocampus after both an immediate shock and after context fear conditioning. Additionally, both shock "alone" and CFC groups show distinct changes in gene expression and translational efficacy. Overall these are interesting studies, and provide another dimension of nuance to some of the work on gene expression and memory, and novel memory-related mechanisms and targets. However, I also think the scholarship as it pertains to accurately portraying previous work, and integrating these findings into the extensive and detailed literature on molecular mechanisms of context fear conditioning. In order to show the relevance and value of these results, it will be important to reframe them in relation to the existing literature. Finally, the results are overinterpreted - the authors are only ruling out one part of the non-associative-experience-dependent transcription and translation occurring (US "alone") but ignoring another part (context exposure). This is ultimately okay - we can't look at everything all the time - but it does need to be acknowledged.

Major concerns:

- I am very confused by the group named "CFC minus shock" throughout the manuscript. Is that context alone? Or do you just mean CFC (context+shock / CFC-shock)? I went with the assumption that it was supposed to refer to the CFC group. In general, the CFC group is named a lot of different things throughout the manuscript: CFC, CFC+shock, CS+US, CFC minus shock (if I'm interpreting that correctly), this needs to be edited for consistency and clarity.
- The authors state that "most studies use naïve animals as a baseline for assessing gene-expressions changes, while only few studies have examined the effect of the US alone, without pairing to context". Although this is reasonably accurate with respect to large scale sequencing, there is a lot of data and a lot of papers showing that cFos and Arc protein levels are elevated after context fear conditioning but not immediate shock (or "shock alone"). This bocy of work should be better represented in this manuscript. And in fact, the data in this paper are consistent with a lot of this work - even though cFos and Arc show increased expression, they do not appear to have increased translation; this mirrors the lack of elevation of cFos protein after immediate shock protocols.
- On this note, are iegs (e.g., cFos) really considered a memory gene (or memory protein)? It is required for memory formation (as is Arc), but it is also activated by a wide range of other stimuli, including footshock stressors.
- One of the interesting and novel aspects of this paper is looking at both translation and transcription - yet there is no detailed discussion of why/how these diverge and what that means for how memory related molecular mechanisms are regulated both at the transcriptional level and the translational level. For example, if transcription of cFos and Arc are increased after footshock alone and after CFC, but translation of these are only increased after CFC, how should we think about translational mechanisms and their importance for specificity of processes, especially for immediate early genes that are regulated by many different external stimuli.

Other concerns and comments:

- It would be ideal to have an identical US in both the "shock alone" and the CFC group. A four second shock is NOT the same as 2 2-second footshocks. I realize that it is not feasible to redo all the sequencing, but this should be acknowledged.
- The name of the "shock alone" group needs to be changed - "Immediate shock" is a better, more accurate description of this group. There is still a context surrounding being

moved to the conditioning room and being placed in a novel box and then receiving a footshock (see the seminal work by Jerry Rudy). And even though there is not strong context fear conditioning there is still learning (But see Bernier, Lacagnina and Drew, 2015, Learn Mem who make a strong argument that animals do learn after immediate shock).

- The limitations of a single time point should also be acknowledged in the discussion.
- There also needs to be some discussion of whether (or not) these findings are consistent with other work on hippocampal gene expression and pathways activated after context fear conditioning.
- The title also feels a little jarring - gene expression is about more than the genes, it's also about the signaling mechanisms, epigenetic modifications, and translational processes that regulate gene expression. Please change this. Even "…memory-related gene expression…" would be better.

**Reviewer 2**

Simbriger et al. apply state-of-the-art global gene expression measurements (transcriptome and ribosome density measurements) to determine which expression changes underlie memory formation due to contextual fear conditioning (CFC). Importantly they relate these changes not only to naïve animals, but also compare the expression changes caused by CFC to the expression changes caused by the aversive unconditioned stimulus (US) alone, which itself is part of the CFC, but in itself does not lead to long-term memory formation. Importantly, the authors did not just look at global expression changes in rodent brain, but looked specifically at expression changes in the dorsal hippocampus, a key brain region for memory formation. The authors indeed find that both CFC and "US alone" lead to pervasive transcriptional and translational changes and interestingly some of the genes, such as Fos and Arc, that have been tightly associated with memory formation upon CFC, show also expression changes upon "US alone", where no long-term memory is established. Upon deeper analysis and subtracting the effect of "US alone", the authors establish a new set of genes that are solely associated with long-term memory formation upon CFC.

To be completely honest, I am not too familiar with the neuroscience field of memory formation and it is hard for me to evaluate the novelty of the findings presented here. From an outsider perspective, these findings seem crucial at it is indeed essential to have the right control for these kind of questions and I do agree that "US alone" is an essential control to add to such studies. Especially in the light of findings presented here that some key genes previously associated with memory formation already show changes upon "US alone". This could potentially have a huge impact on the field.

From the perspective of the gene expression field, especially in regards to post-transcriptional gene expression regulation, a field I am much more familiar with, the authors present a great dataset. Obtaining matched transcriptome and ribosome footprinting data directly from the dorsal hippocampus of such quality is extremely challenging, even more so that the authors did so under 3 different experimental conditions. The authors are also very cautious about their conclusions and do largely not overinterpret the data, which I very much appreciate. Therefore I think that publication of the presented data would be of big interest to the gene expression field and most likely also to the neuroscience field (again, the reason I am cautious about that statement is that I do not regard myself sufficiently entangled in the field to be completely up-to-date on the literature). I do have a few suggestions (mainly smaller ones) that I think would be great to be addressed before acceptance. All of these "issues" can be resolved with some additional analysis and/or some additional explanations.

Major point:

1. The finding that there is a significant overlap in gene expression changes between CFC and "US only" would seem extremely important for the field (especially if some of these genes are used as markers of memory formation). However, it also is important to see if the amplitude of the response is the same and therefore I would not just suggest a qualitative comparison between the conditions (meaning which genes are significantly changed in each condition alone and which genes in both), but also a direct quantitative comparison between CFC and "US alone". It might be that there is a number of the same genes induced under both experimental settings, but that the strength of the response is still significantly different.

Minor points:

2. Page 4; lines 74 to 77: "We observed that in all examined tissues, there is a trend for reduced TE for ribosomal protein-coding mRNAs, as compared to all proteins and to mitochondrial ribosomal proteins (Sup. Fig. 1c) and that the decrease seen in hippocampus was comparable to other tissues."

   About the low TE in ribosomal proteins - did the authors also compare to other genes expressed at similar high levels and the same mRNA length (as ribosomal proteins are rather small), as only few ribosomes could theoretically fit on the mRNA and production could be guided by high elongation to initiation rate.

3. Page 4; lines 85 and 86: "…… fourth by the r2 of RPKM between biological replicates (n=2) for the three experimental groups, which is >0.98 for both footprints and total mRNA (Sup. Fig. 1d)."

   What is the correlations of delta footprints or delta RNA levels between the replicates. The high reproducibility between replicates as often shown for RNA-seq and ribosomal footprints data is expected due to high dynamic range (high expressed genes will stay high expressed and low expressed will stay low expressed (even in very noisy data)), but the question is: can you reproducible see for example a two fold change between conditions? Would be great to add these plots at least to the Supplemental Material.

4. Page 4; lines 88 to 90:"This is evidenced by the low correlation (Pearson R2<0.45) of log2 TE or RPKM normalised to homecage, for translation and transcription respectively (Fig. 1c, d)."

   The same as above. Would need to know how reproducible the delta changes are between replicates in order to assess how different really the two stimuli conditions are. Otherwise the R2 of 0.45 is hard to evaluate.

5. Page 7; lines 153 to 155: "These results suggest that contextual memory in CFC is acquired following translational control of a subset of genes, chiefly via 5' UTR mechanisms (and thus possibly involving translation initiation control)."

   That conclusion is a bit of a leap. Could be, but for sure not true for all and also the signal is not very strong. Again, cannot be excluded and the authors did write "suggest", but I would suggest to remove the "chiefly" as there is no strong evidence for this in my opinion. The 5'UTR might have a role, but "chiefly" cannot be concluded from the data presented.

6. Page 8; lines 160 to 164 and legend for Figure 3b: "[Fig. 3b; top; main effect of motif type F (3, 9) = 16.37; p=0.0005], as compared to all other groups (p<0.0001) and detected terminal oligopyrimidine tract containing (TOP) mRNAs in shock only DTGs, but not in CFC (Fig. 3b; top). While we observed a significant effect for motif type: F (8, 24) = 102.8; p<0.0001, there were no significant changes in 3' UTR motifs between CFC and shock only DTGs [effect of group: F (3, 24) = 9.376, p>0.999 (Fig. 3b; bottom)]."

I am not sure what the authors mean with "F" here? A general type of motif, the underlying statistics? Also what are the numbers in the parenthesis? I am sorry that I probably missed it, but I also did not find more information about that in materials and methods. I probably should know what it means, but I do not, and could imagine that this could also confuse a few readers. Could you maybe add some more information about the statistics applied, etc.?

7. Page 10, line 209 (Figure Legend): "Continuous UV absorbance at 254 nm of lysates resolved over a 5-15% sucrose gradient."

   It probably should say "50%" instead of "15%"?

8. US alone and shock only, please write as "US alone" and "shock only". Otherwise it made me read the sentences twice (especially in the beginning) where these combinations are used.

9. Figure 3a: would prefer box plots - easier to interpret.

## 1st Author Response Letter

**Reviewer 1**

*Major concerns:*

- *I am very confused by the group named "CFC minus shock" throughout the manuscript. Is that context alone? Or do you just mean CFC (context+shock / CFC- shock)? I went with the assumption that it was supposed to refer to the CFC group. In general, the CFC group is named a lot of different things throughout the manuscript: CFC, CFC+shock, CS+US, CFC minus shock (if I'm interpreting that correctly), this needs to be edited for consistency and clarity.*

Response: We acknowledge, that the chosen terminology can lead to confusion and have added more detail on individual groups as well as making the naming consistent throughout the text. The corrected group names are now: “homecage”, “immediate shock”, and “CFC” and are italicized throughout the text. We have also addressed this in response to reviewer #2 below (Point 8.).

- *The authors state that "most studies use naïve animals as a baseline for assessing gene-expressions changes, while only few studies have examined the effect of the US alone, without pairing to context". Although this is reasonably accurate with respect to large scale sequencing, there is a lot of data and a lot of papers showing that cFos and Arc protein levels are elevated after context fear conditioning but not immediate shock (or "shock alone"). This body of work should be better represented in this manuscript. And in fact, the data in this paper are consistent with a lot of this work - even though cFos and Arc show increased expression, they do not appear to have increased translation; this mirrors the lack of elevation of cFos protein after immediate shock protocols.*

Response: We agree with the reviewer that there are studies showing increased cFos/Arc levels following CFC but not in the “immediate shock” condition. We now cite many of these papers and better explain our main point that this control condition is often lacking in genome-wide gene expression studies. This is summarised in supplementary Table 1.

- *On this note, are iegs (e.g., cFos) really considered a memory gene (or memory protein)? It is required for memory formation (as is Arc), but it is also activated by a wide range of other stimuli, including footshock stressors.*

Response: As correctly indicated by the reviewer, previous studies have shown that cFos is induced by a variety of stimuli and its conditional deletion in neurons1 or knockdown using ASO2,3 resulted in contextual fear memory impairment. The biological role and downstream effectors of cFos in the brain remain elusive. In neurons, cFos expression is stimulated by cAMP and intracellular calcium through the activation of the CREB/CRE complex. The Fos protein, encoded by c-fos gene, dimerizes with Jun family transcription factors to build up the transcription factor AP-1. AP-1 downstream genes and their roles in synaptic plasticity and memory formation are not well characterized. This is in stark contrast to other IEGs, such as *homer1a* and *arc* that are known to encode proteins that have a direct effect on the synapses. In the discussion section of the manuscript, we now better describe the known roles of Arc in experience-dependent dendritic remodeling and hitherto enigmatic functions of cFOS in memory formation.

- *One of the interesting and novel aspects of this paper is looking at both translation and transcription - yet there is no detailed discussion of why/how these diverge and what that means for how memory related molecular mechanisms are regulated both at the transcriptional level and the translational level. For example, if transcription of cFos and Arc are increased after footshock alone and after CFC, but translation of these are only increased after CFC, how should we think about translational mechanisms and their importance for specificity of processes, especially for immediate early genes that are regulated by many different external stimuli.*

Response: We apologise for this omission and agree with the reviewer that we need to better highlight this aspect of our work. In the discussion section (last 2 paragraphs) of the revised manuscript, we now mention that transcription is often uncoupled from translation, describe translation buffering and also speculate about the contribution of these mechanisms for specificity of processes underlying memory formation. We have also addressed part of this point in response to Reviewer #2 below by generating Sup. Fig. 4, comparing transcriptional and translational modulation of the top gene targets identified in Fig.1 and validated in Fig.4.

*Other concerns and comments:*

- *It would be ideal to have an identical US in both the "shock alone" and the CFC group. A four second shock is NOT the same as 2 2-second footshocks. I realize that it is not feasible to redo all the sequencing, but this should be acknowledged.*

Response: We agree with the reviewer that having identical US in both “immediate shock” and CFC groups would be an ideal experimental design. The rationale for the “immediate shock” condition is to test the effect of footshock without allowing mice to associate it with the context. Since our CFC US protocol consists of two 2 s footshocks separated by a 30 s interval, the mice have 30 s between two footshocks to make an association between footshock and the environment. To avoid this situation, which would diminish the purpose of this control condition, we opted to deliver one 4 s footshock. According to this protocol, mice are given one 4 s footshock immediately after being placed in the chamber and then removed from the chamber upon footshock termination. This protocol gives mice a very short period of time to experience the environment and minimize the association between context and the footshock. As suggested by the reviewer, in the revised version of the manuscript we clearly indicate that footshock stimulation protocol for “immediate” differs from the CFC condition (pairing of context to footshock).

- *The name of the "shock alone" group needs to be changed - "Immediate shock" is a better, more accurate description of this group. There is still a context surrounding being moved to the conditioning room and being placed in a novel box and then receiving a footshock (see the seminal work by Jerry Rudy). And even though there is not strong context fear conditioning there is still learning (But see Bernier, Lacagnina and Drew, 2015, Learn Mem who make a strong argument that animals do learn after immediate shock).*

Response: We thank the reviewer for their insightful comment and have changed the name of the experimental groups accordingly throughout the manuscript. We have also addressed the point regarding immediate shock and context in the previous remark by the reviewer above and in response to Reviewer #2.

- *The limitations of a single time point should also be acknowledged in the discussion.*

Response: We have now amended this in the discussion and mention the Cho et al. paper, which uses several timepoints.

- *There also needs to be some discussion of whether (or not) these findings are consistent with other work on hippocampal gene expression and pathways activated after context fear conditioning.*

Response: We agree and have added this important point in the discussion with references to previous relevant work, part of which is also included in Sup. Table 1.

- *The title also feels a little jarring - gene expression is about more than the genes, it's also about the signaling mechanisms, epigenetic modifications, and translational processes that regulate gene expression. Please change this. Even "...memory- related gene expression..." would be better.*

Response: We have now changed the title according to the reviewer’s suggestion (line 1) to read:

> “Uncovering memory-related gene expression in contextual fear conditioning using ribosome profiling”

**Reviewer 2**

*Major point:*

1. *The finding that there is a significant overlap in gene expression changes between CFC and "US only" would seem extremely important for the field (especially if some of these genes are used as markers of memory formation). However, it also is important to see if the amplitude of the response is the same and therefore I would not just suggest a qualitative comparison between the conditions (meaning which genes are significantly changed in each condition alone and which genes in both), but also a direct quantitative comparison between CFC and "US alone". It might be that there is a number of the same genes induced under both experimental settings, but that the strength of the response is still significantly different.*

Response: We thank the reviewer for their constructive feedback and have now added a new figure (Supplementary Figure 4) summarizing the expression levels of the genes that were found to overlap in the two experimental groups of Fig. 1. We have also carried out statistical analysis for each gene (included in the appendix of this letter).

A direct comparison of expression levels of genes in both the “immediate shock” and the “CFC” condition (normalized to “homecage” levels) showed that the amplitude of the response is comparable for most genes (Sup. Fig. 4 and appendix statistics).

However, from the Ribosome profiling data and RT-qPCR validation (Fig. 4), we do see that while there are some genes that are activated only in “CFC” and not in “immediate shock” (translationally or transcriptionally), there may be a difference in the amplitude of their activation. For example, while Arc, Fos and Egr2 are detected at similar levels in the “CFC” and “immediate shock” conditions with RT-qPCR (Fig. 4c) and also as predicted by ribosome profiling (Fig. 1 and Sup. Fig. 4), we observed for Rsrp1 a clear trend (not significant p=0.06; appendix statistics), suggesting a possible lower induction in “CFC” than in “immediate shock” (Sup. Fig. 4). Together, these data reveal that there may be genes which are activated in both “CFC” and “immediate shock” conditions, but the magnitude of their activation is attenuated in CFC. Further investigation of our proposed targets is however required, and there may be several gene-expression modules to separate “CFC” from “immediate shock”. We have now added this in the discussion section.

*Minor points:*

2. *Page 4; lines 74 to 77: "We observed that in all examined tissues, there is a trend for reduced TE for ribosomal protein-coding mRNAs, as compared to all proteins and to mitochondrial ribosomal proteins (Sup. Fig. 1c) and that the decrease seen in hippocampus was comparable to other tissues."*

   *About the low TE in ribosomal proteins - did the authors also compare to other genes expressed at similar high levels and the same mRNA length (as ribosomal proteins are rather small), as only few ribosomes could theoretically fit on the mRNA and production could be guided by high elongation to initiation rate.*

Response: We thank the reviewer for their comment and have added information to Sup. Fig. 1c detailing the TE levels of genes with similar length to ribosomal protein mRNAs. We also describe this methodology in the methods section.

3. *Page 4; lines 85 and 86: "...... fourth by the r2 of RPKM between biological replicates (n=2) for the three experimental groups, which is >0.98 for both footprints and total mRNA (Sup. Fig. 1d)."*

   *What is the correlations of delta footprints or delta RNA levels between the replicates. The high reproducibility between replicates as often shown for RNA-seq and ribosomal footprints data is expected due to high dynamic range (high expressed genes will stay high expressed and low expressed will stay low expressed (even in very noisy data)), but the question is: can you reproducible see for example a two fold change between conditions? Would be great to add these plots at least to the Supplemental Material.*

4. *Page 4; lines 88 to 90: "This is evidenced by the low correlation (Pearson R2<0.45) of log2 TE or RPKM normalised to homecage, for translation and transcription respectively (Fig. 1c, d)."*

   *The same as above. Would need to know how reproducible the delta changes are between replicates in order to assess how different really the two stimuli conditions are. Otherwise the R2 of 0.45 is hard to evaluate.*

Response: We thank the reviewer for the constructive comments in points 3 and 4. We have provided data in the original submission to support the high quality of our RNAseq libraries (Sup. Fig. 2). We agree that the best way to examine reproducibility is by direct comparison of biological replicates. However, it is not possible to calculate delta footprints or delta mRNA levels, because the individual biological replicates are not connected (e.g. “homecage”

biological replicate 1 or 2 are not linked to the "immediate shock" biological replicate 1 or 2 groups). Thus, to calculate the Immediate Shock/Homecage and CFC/Homecage groups presented in Fig. 1 and Sup. Fig. 1, we used the average values from the biological replicates. However, to further investigate intergroup reproducibility, we carried out Principal Components Analysis (new Sup. Fig. 3) for each of the biological replicates. The proximity of components between groups further supports our claim for high reproducibility; dotted lines on the graph. Moreover, there is a clear distinction between the 3 experimental groups (homecage, immediate shock, and CFC) for both translational efficiency and transcription (Sup. Fig. 3a, b). We would also like to highlight the fact that as described in methods, we used 2 biological replicates, each containing dorsal hippocampi from 5-6 animals. This experimental design was selected due to starting material limitations in the ribosome profiling protocol, whereby we needed to pool several animals for subsequent nuclease treatment (300 ng minimum total RNA and small size of dorsal hippocampus). While this factor may increase the variability in our measurements of biological replicates, the QC we carried out along with the new PCA support the reproducibility between replicates.

5. *Page 7; lines 153 to 155: "These results suggest that contextual memory in CFC is acquired following translational control of a subset of genes, chiefly via 5' UTR mechanisms (and thus possibly involving translation initiation control)."*

   *That conclusion is a bit of a leap. Could be, but for sure not true for all and also the signal is not very strong. Again, cannot be excluded and the authors did write "suggest", but I would suggest to remove the "chiefly" as there is no strong evidence for this in my opinion. The 5'UTR might have a role, but "chiefly" cannot be concluded from the data presented.*

Response: We agree with the reviewer and have amended the sentence as suggested to now read:

> "These results suggest that contextual memory in CFC is acquired following translational control of a subset of genes, via 5' UTR mechanisms (and thus possibly involving translation initiation control)."

6. *Page 8; lines 160 to 164 and legend for Figure 3b: "[Fig. 3b; top; main effect of motif type F (3, 9) = 16.37; p=0.0005], as compared to all other groups (p<0.0001) and detected terminal oligopyrimidine tract containing (TOP) mRNAs in shock only DTGs, but not in CFC (Fig. 3b; top). While we observed a significant effect for motif type: F (8, 24) = 102.8; p<0.0001, there were no significant changes in 3' UTR motifs between CFC and shock only DTGs [effect of group: F (3, 24) = 9.376, p>0.999 (Fig. 3b; bottom)]."*

   *I am not sure what the authors mean with "F" here? A general type of motif, the underlying statistics? Also what are the numbers in the parenthesis? I am sorry that I probably missed it, but I also did not find more information about that in materials and methods. I probably should know what it means, but I do not, and could imagine that this could also confuse a few readers. Could you maybe add some more information about the statistics applied, etc.?*

Response: We have removed the detailed statistical results from the main text and moved them into the respective figure legend. Details of the standard statistical tests used are summarized in the Methods section "Statistical Analysis". Results of these statistical tests are compiled in Supplementary Table 5. The F-test or F-value is a statistical value computed for each hypothesis in a two-way ANOVA and is defined by the ratio of the mean square for each main effect to the residual mean square. The F-ratios are used to compute p-values and if the null hypothesis is not true, F is likely to be greater than 1.0, while an F-ratio close to 1.0 may indicate a true null hypothesis.

7. *Page 10, line 209 (Figure Legend): "Continuous UV absorbance at 254 nm of lysates resolved over a 5-15% sucrose gradient."*

   *It probably should say "50%" instead of "15%"?*

Response: We thank the reviewer for noticing this typographical error and have now corrected it to read 50%.

8. *US alone and shock only, please write as "US alone" and "shock only". Otherwise it made me read the sentences twice (especially in the beginning) where these combinations are used.*

Response: We agree with the reviewer about the terminology being confusing and have italicized the names of the experimental groups throughout the text for the reader's ease and also have used consistent names for the groups throughout the manuscript. This issue was also addressed in response to Reviewer #1, point 1 under "major concerns".

9. *Figure 3a: would prefer box plots - easier to interpret.*

Response: We have now changed Fig. 3a to show boxplots along with all experimental points.

## 2nd Decision Letter

Dear Dr. Gkogkas,

Thank you for submitting your manuscript to Progress in Neurobiology. We have received comments from reviewers on your manuscript. Your paper should become acceptable for publication pending suitable minor revision and modification of the article in light of the appended reviewer comments.

When resubmitting your manuscript, please carefully consider all issues mentioned in the reviewers' comments, outline every change made point by point, and provide suitable rebuttals for any comments not addressed.

To submit your revised manuscript go to https://www.editorialmanager.com/proneu/ and log in as an Author where you will see a menu item called 'Submission Needing Revision'.

Please resubmit your manuscript by Jul 04, 2020.

We look forward to receiving your revised manuscript.

Kind regards,

Laura Colgin
Associate Editor

Sabine Kastner
Editor-in-Chief
Progress in Neurobiology

**Comments from the Editors and Reviewers:**

**Reviewer 1**

Overall, the authors have responded in detail to my comments. I remain enthusiastic about this manuscript. I have a few minor comments, reiterating a little on one point from before.

The authors still make strong arguments that (1) immediate shock does not result in memory, and (2) that other studies have failed to use this as a control.

The argument that immediate shock doesn't result in memory formation, has been effectively challenged, see: Bernier, Lacagnina, & Drew (2015) Learning & Memory 22:31-38. One should be careful of conflating a lack of any memory formed, and a lack of formation or expression of a context fear memory. (2) immediate shock is a widely used control. This is mostly toned down in the body of the paper, but the abstract would be stronger with a focus on the substantial strengths of the current study.

In addition, please specify that the animals used in this study are males. I know that it is noted in the methods, but it should be clear throughout the manuscript that this work is on male mice.

On line 116 the authors say "CFC without immediate shock"... is this a typo? If not please restate what you mean here.

**Reviewer 2**

I want to congratulate the authors for providing an improved revised manuscript. They answered most of my "concerns". In general, I fully support publication, but have a few minor points that the authors could maybe address before final acceptance. I also want to apologize that a few of these I did not bring up in the first round of revision.

The minor points are the following:

1. In the response to the reviewers about looking at the correlation of CHANGES between replicates the authors answered: "However, it is not possible to calculate delta footprints or delta mRNA levels, because the individual biological replicates are not connected (e.g. "homecage" biological replicate 1 or 2 are not linked to the "immediate shock" biological replicate 1 or 2 groups)."

   Although I technically agree with the authors, they can still do it by randomly pairing, which is for such an analysis completely ok in my opinion. The main reason for me asking for such an analysis is to put the R2 of 0.45 for TEs between "CFC" and "US only" into context (in that sense the authors should actually also show the replicate correlations of delta TE). The authors write that this low R2 indicates strong differences in translational regulation, but it is hard to decipher how much is due to noise. Yes the R2 of the rpkms looks very good, but as I pointed out that could be strongly due to high dynamic range of the data. By randomly pairing the replicates they can generate such an R2 and they can test even all random pairs to give a range of R2 values between replicates. Another way to look at it is to do a general 6 times 6 matrix of R2 between the rpkms of all footprinting experiments and RNA-seq separately and TE values (so CTL1

to itself and CTL2; to US only 1 and 2; to CFC 1 and 2; etc.) - that gives a good feeling if the replicate changes are smaller than the conditions changes. I absolutely assume this to be the case, but I still think it is nice to be presented. The PC analysis confirms that partly, but in all honesty the PCA separation between the conditions for the TE values, although present, it is not too strong, like for example the one for RNA-seq data (one issue could be that when I opened the word suppl file, some characters in the graphs did not parse correctly and showed random symbols, so I could not read every sample (or axis) - I think I could determine correctly what is what, but can't be 100% sure - see below more about that). Even if it turns out that the replicate correlations are not so much better than the condition correlations, it will help to interpret the results and the authors did do rigorous statistical testing afterwards to call the significant genes.

2. I did not pay attention to that before, but in Figure 1C and 1D, it is a bit weird that all the significantly changed genes actually are more towards the inside of the scatter (so lower fold changes) and not at the outside where the extreme changes are. I guess that means that the extreme changes were not very consistent? Were these values really so inconsistent between the replicates or was it just that they were for example strongly changed in both but the single log2 ratio values were too different to be called significant on two replicates only? Could the authors maybe comment on that? I actually wanted to look this up myself, but could not find any table with all the rpkm for FP, RNA-seq and TE values. The link to the raw data and where I was hoping to get these table(s) did not work for me. I think such an overview table with all normalized values, the calculated TE values and associated p-values would be extremely useful as a supplemental online table. It would be great if that could be added by the authors.
3. The transcription correlation in 1D is considerably worse than the TE correlation in 1C, which is surprising as the transcription goes into the calculation of the TE values. What is the correlation of translation directly? Is it considerably better? Do you see basically compensation by translation?
4. I had some problem with a few of the supplemental files and links. As written above when I used the link to get to the raw data it did not work. I could not open any the supplemental downloaded Excel files (Suppl. Tab. 2 and 4) - my Excel program told me the files are "corrupted". Opening the supplemental materials, which is a world file, Supplemental Figure 3 had weird special characters (e.g. all replicate 2 sample description were weird special characters as well as the y-axis). I have a PC and it could be that this is the problem. Maybe saving it as a pdf could alleviate that problem?
5. Row 515 and Figure 1: the authors write about the cutoff used "0.6671.5". Sorry to be nitpicky, but for me that means ratio is either bigger than 0.667 or 1.5, but I guess the authors meant smaller than 0.667 or bigger 1.5, so I would write "0.667>ratio>1.5".
6. Row 559 - the referral to Suppl. Table 4. Isn't it supposed to be Suppl. Table 5?
7. Figure 4 between panels b and c: the graphic depicted CFC only and immediate shock genes. The graphic for immediate shock is different than used in Figures 2b and 3b, where this gene group is only depicted by the grew circle with "US" in it? Is this now another definition in Figure 4? If not please use the same graphic.

## 2nd Author Response Letter

**Reviewer 1**

*Overall, the authors have responded in detail to my comments. I remain enthusiastic about this manuscript. I have a few minor comments, reiterating a little on one point from before.*

*The authors still make strong arguments that (1) immediate shock does not result in memory, and (2) that other studies have failed to use this as a control.*

*The argument that immediate shock doesn't result in memory formation, has been effectively challenged, see: Bernier, Lacagnina, & Drew (2015) Learning & Memory 22:31-38. One should be careful of conflating a lack of any memory formed, and a lack of formation or expression of a context fear memory.*

Response: We thank the reviewer for their comment and would like to stress that the work by Bernier *et al*[1] very nicely shows the effects of different pre- and postshock intervals on the expression of contextual fear memory. Moreover, they show that preshock intervals less than 10 s, which most closely resembles our immediate shock condition, failed to produce robust fear memories when tested 24 h later. Several other studies, conducted in different rat and mouse strains, show similar results of immediate shock paradigms or very short pre-exposure intervals being insufficient to produce robust fear memories[2–5]. Furthermore, our own data (Fig 1A) show insignificant levels of freezing in *immediate shock* compared to *homecage*, for our behavioral paradigm. To address the reviewer's concern, we stress the importance of pre- and postshock intervals in the discussion and cite Bernier *et al*.

*(2) Immediate shock is a widely used control. This is mostly toned down in the body of the paper, but the abstract would be stronger with a focus on the substantial strengths of the current study.*

Response: We agree with the reviewer, that immediate shock is a widely used control in behavioral studies, however, as we stress in our manuscript and we summarized in Supplementary Table 1, immediate shock has been underused as a control in genome-wide expression studies, such as ours.

*In addition, please specify that the animals used in this study are males. I know that it is noted in the methods, but it should be clear throughout the manuscript that this work is on male mice.*

Response: We now state in the abstract and the text that all animals used in our study were adult males.

*On line 116 the authors say "CFC without immediate shock"... is this a typo? If not please restate what you mean here.*

Response: We apologize for being unclear and now state on line 111 that "*CFC* minus *immediate shock*" refers to all genes regulated in the *CFC* group after removing any genes overlapping with genes regulated in the *immediate shock* group.

**Reviewer 2**

*I want to congratulate the authors for providing an improved revised manuscript. They answered most of my "concerns". In general, I fully support publication, but have a few minor points that the authors could maybe address before final acceptance. I also want to apologize that a few of these I did not bring up in the first round of revision.*

*The minor points are the following:*

1. *In the response to the reviewers about looking at the correlation of CHANGES between replicates the authors answered: "However, it is not possible to calculate delta footprints or delta mRNA levels, because the individual biological replicates are not connected (e.g. "homecage" biological replicate 1 or 2 are not linked to the "immediate shock" biological replicate 1 or 2 groups)."*

   *Although I technically agree with the authors, they can still do it by randomly pairing, which is for such an analysis completely ok in my opinion. The main reason for me asking for such an analysis is to put the R2 of 0.45 for TEs between "CFC" and "US only" into context (in that sense the authors should actually also show the replicate correlations of delta TE). The authors write that this low R2 indicates strong differences in translational regulation, but it is hard to decipher how much is due to noise. Yes the R2 of the rpkms looks very good, but as I pointed out that could be strongly due to high dynamic range of the data. By randomly pairing the replicates they can generate such an R2 and they can test even all random pairs to give a range of R2 values between replicates. Another way to look at it is to do a general 6 times 6 matrix of R2 between the rpkms of all footprinting experiments and RNA-seq separately and TE values (so CTL1 to itself and CTL2; to US only 1 and 2; to CFC 1 and 2; etc.) - that gives a good feeling if the replicate changes are smaller than the conditions changes. I absolutely assume this to be the case, but I still think it is nice to be presented. The PC analysis confirms that partly, but in all honesty the PCA separation between the conditions for the TE values, although present, it is not too strong, like for example the one for RNA-seq data (one issue could be that when I opened the word suppl file, some characters in the graphs did not parse correctly and showed random symbols, so I could not read every sample (or axis) - I think I could determine correctly what is what, but can't be 100% sure - see below more about that). Even if it turns out that the replicate correlations are not so much better than the condition correlations, it will help to interpret the results and the authors did do rigorous statistical testing afterwards to call the significant genes.*

Response: We thank the reviewer for their insightful comment and have added the 6 by 6 R2 correlation matrices of footprints/total mRNA/TE for all replicates and experimental conditions to Supplementary Figure 2 (panel e) in response.

2. *I did not pay attention to that before, but in Figure 1C and 1D, it is a bit weird that all the significantly changed genes actually are more towards the inside of the scatter (so lower fold changes) and not at the outside where the extreme changes are. I guess that means that the extreme changes were not very consistent? Were these values really so inconsistent between the replicates or was it just that they were for example strongly changed in both but the single log2 ratio values were too different to be called significant on two replicates only? Could the authors maybe comment on that? I actually wanted to look this up myself, but could not find any table with all the rpkm for FP, RNA-seq and TE values. The link to the raw data and where I was hoping to get these table(s) did not work for me. I think such an overview table with all normalized values, the calculated TE values and associated p-values would be extremely useful as a supplemental online table. It would be great if that could be added by the authors.*

Response: The software we use to call significant genes in both transcription (z-score analysis) and translation (Xtail) is very stringent on what is called a “significant gene” and filters out extreme changes in the data. Based on the fact that we only have two replicates,

this may have caused the most extreme changes to get cut from the list of significant genes. A bigger sample size would be preferable but was not a possibility for us at the time.

We apologize for the difficulties with the downloadable raw data and have made sure that the data is now easily available on either Mendeley or as part of the downloadable Supplementary data.

3. *The transcription correlation in 1D is considerably worse than the TE correlation in 1C, which is surprising as the transcription goes into the calculation of the TE values. What is the correlation of translation directly? Is it considerably better? Do you see basically compensation by translation?*

Response: We thank the reviewer for raising this very interesting question. The timepoint we chose for profiling is 20 min after behavioral training, which is the earliest timepoint at which one can usually see translation. As transcription usually shows greater changes already at an earlier timepoint, we would expect it to show lower correlation compared to TE. Furthermore, it could be possible that the divergence that we observe is due to translational buffering mechanisms that remain to be elucidated.

4. *I had some problem with a few of the supplemental files and links. As written above when I used the link to get to the raw data it did not work. I could not open any the supplemental downloaded Excel files (Suppl. Tab. 2 and 4) - my Excel program told me the files are "corrupted". Opening the supplemental materials, which is a world file, Supplemental Figure 3 had weird special characters (e.g. all replicate 2 sample description were weird special characters as well as the y-axis). I have a PC and it could be that this is the problem. Maybe saving it as a pdf could alleviate that problem?*

Response: We see how this may cause problems for some users and will make sure to provide supplementary information in transferrable formats.

5. *Row 515 and Figure 1: the authors write about the cutoff used "0.667<ratio>1.5". Sorry to be nitpicky, but for me that means ratio is either bigger than 0.667 or 1.5, but I guess the authors meant smaller than 0.667 or bigger 1.5, so I would write "0.667>ratio>1.5".*

Response: We agree with the reviewer and have amended the information in the figure and the figure legend to read “0.667 > ratio > 1.5”.

6. *Row 559 - the referral to Suppl. Table 4. Isn't it supposed to be Suppl. Table 5?*

Response: We refer to Suppl. Table 4, summarizing the published data used for the comparison in this figure.

7. *Figure 4 between panels b and c: the graphic depicted CFC only and immediate shock genes. The graphic for immediate shock is different than used in Figures 2b and 3b, where this gene group is only depicted by the grew circle with "US" in it? Is this now another definition in Figure 4? If not please use the same graphic.*

Response: We acknowledge how the use of the graphics may have caused some confusion and have added a more detailed description of the groups in the figure legend.
